# A di-nuclear μ-oxido rhenium(vi) complex: from deep purple to catalytic applications

**DOI:** 10.1039/d6dt00039h

**Published:** 2026-02-27

**Authors:** Tobias A. Doliner, Peter E. Hartmann, Gernot Oberwinkler, Ferdinand Belaj, Antoine Dupé, A. Daniel Boese, Jörg A. Schachner

**Affiliations:** a Institute of Chemistry, University of Graz Schubertstr. 1 8010 Graz Austria joerg.schachner@uni-graz.at

## Abstract

In this manuscript, we report on the formation and isolation of the di-nuclear rhenium(vi) µ-oxido bridged complex {[ReOCl_2_(L1)]}_2_(µ-O) (1), which we nick-named “deep purple” due to its intense purple color both in the solid state as well as in solution, and its surprising catalytic activity in oxyanion reduction. Complex 1 was obtained as the unintended product in an attempt to synthesize oxidorhenium(v) complex [ReOCl(L1)_2_], using the *O*,*N*-bidentate phenol-dimethyloxazoline ligand HL1, equipped with two electron-donating *tert*-butyl groups on the phenol ring. Single crystal X-ray diffraction analysis finally revealed the surprising µ-oxido structure of 1, with two paramagnetic Re(vi) centers and a mixed *cis*/*trans*-chlorido arrangement on the two Re centers*. Via* a targeted synthesis in cyclohexane at 60 °C under ambient conditions, high yields of over 90% of 1 were obtained. To explain the source of the highly stereoselective formation of 1, its formation was examined by DFT calculations at the B3LYP-D3BJ/dhf-TZVPP@B3LYP-D3BJ/dhf-SVP level of theory, with solvent effects included *via* the COSMO model. In contrast to isoelectronic µ-oxido bridged Mo(v) complexes, 1 proved to be highly active in catalytic oxyanion reduction, namely perchlorate and nitrate reduction. With a catalyst loading of 5 mol% of 1, a conversion of 90% was observed for perchlorate. These findings highlight that in rhenium chemistry, µ-oxido bridged complexes like 1 still show promising catalyst activities for oxyanion reduction and potentially also other redox transformations.

## Abstract 2

From lab curiosity to a novel Re(vi) oxyanion reduction catalyst, we proudly present deep purple.

## Graphical abstract

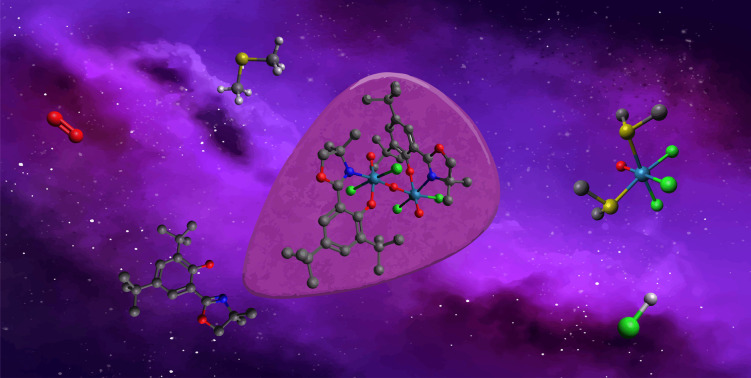

## Introduction

Oxido ligands (O^2−^) play a crucial role in stabilizing high oxidation states in metal complexes, forming either terminal or bridging bonds between metal centers.^1^ In biological systems, oxido ligands are essential for enzymatic function, appearing for example in molybdenum cofactors and iron-containing enzymes.^2^ In rhenium chemistry, three primary oxido moieties exist, {ReO}, {ReO_2_} and {ReO_3_}, each displaying unique structural and electronic characteristics.^3^ Di-nuclear µ-oxido bridged species, such as [Cp*Re_2_O_3_Cl_2_] and [Re_2_O_4_Me_4_], are particularly stable due to strong metal-oxido interactions.^4^ These complexes can form either through a non-reductive mechanism, where oxido bridges result from controlled ligand rearrangements, or a reductive mechanism in which oxygen scavengers like triphenylphosphine facilitate oxido bridge formation.^5^

In rhenium complexes, particularly those that are µ-oxido bridged, superexchange interactions play a significant role in their magnetic properties and are essential for their characterization.^6,7^ Superexchange, as described by Kramers^8^ and Anderson,^9^ involves magnetic coupling between transition metal centers through intervening non-magnetic atoms, such as the oxido groups bridging the metal centers. This interaction is especially pertinent to complex {[ReOCl_2_(L1)]}_2_(µ-O) (1) as it allows the study of the complex *via* nuclear magnetic resonance (NMR) spectroscopy by mitigating the complications typically associated with paramagnetic effects.^10–12^ The superexchange mechanism mediates the magnetic coupling between the two paramagnetic Re(vi) centers of complex 1 through the bridging oxygen atom, reducing paramagnetic relaxation and thus enabling detailed NMR analysis of “deep purple” 1.

**Fig. 1:**
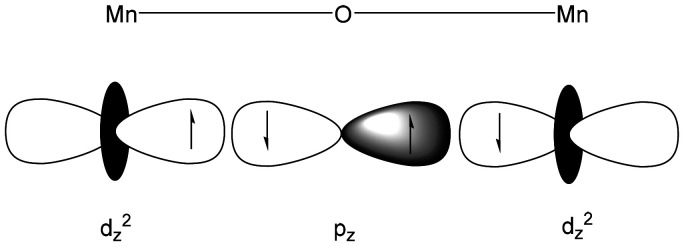
Demonstration of the superexchange interaction in {Mn_2_O}, where orbital overlap influences the magnetic properties and the alignment of electron spins.^19^

The Goodenough–Kanamori rules^13–15^ are useful for predicting the nature of the magnetic alignment (ferromagnetic or antiferromagnetic) arising from superexchange interactions, based on orbital overlap and the electron configurations of the metal centers (Fig. 1).^16,17^ In the case of transition-metal oxides and µ-oxido bridged rhenium complexes, these interactions also influence their conductivity properties, as aligned spins can either enhance or impede electrical conductivity.^18^

Oxygen atom transfer (OAT) reactions play a crucial role in both biological and industrial processes, facilitating the controlled and selective transfer of oxygen atoms between molecules. In nature, enzymes such as cytochrome P450 perform OAT to drive oxidative transformations, while industrial applications utilize similar principles for processes like propylene oxide synthesis.^20,21^ Rhenium has emerged as a particularly promising metal for OAT catalysis due to its ability to adopt multiple stable oxidation states, particularly Re(v) and Re(vii),^22,23^ which mirror those of well-studied Mo(iv/vi)^24,25^ and W(iv/vi)^25^ catalysts. Its strong metal-oxido bonds, combined with its ability to participate in both primary and secondary OAT mechanisms, makes rhenium highly effective for oxygen transfer reactions. Prior work by Abu-Omar *et al.*^23^ demonstrated that oxidorhenium(v) complexes can facilitate perchlorate reduction *via* OAT, highlighting the potential of rhenium-based catalysts in environmental applications.^26,27^

Perchlorate (ClO_4_^−^) contamination has emerged as a critical environmental issue due to its remarkable persistence in aquatic systems and its interference with human thyroid hormone biosynthesis through competitive inhibition of iodide uptake, leading to metabolic and developmental disorders.^28–30^ Highly soluble and kinetically stable, perchlorate spreads easily in groundwater and has already been detected in drinking water, soil, and food products.^31,32^ Industrial activities, particularly those involving rocket fuel, ammunitions and explosives, have significantly contributed to its presence in the environment.^33,34^ Due to its interference with thyroid function, regulatory agencies have proposed strict exposure limits, though enforceable federal standards remain absent in the U.S.^35,36^

Existing remediation methods, such as ion exchange and electrodialysis, effectively remove perchlorate but do not degrade it, leading to secondary waste concerns. Bioremediation, though promising, requires stringent redox control and faces public resistance.^26^ Catalytic reduction offers an alternative, but the strength of the Cl–O bonds (∼250 kJ mol^−1^) presents a significant challenge.^37^ Early transition-metal-based reduction systems required extreme conditions,^38,39^ while more recent efforts have explored rhenium catalysts for oxygen atom transfer (OAT).^23^ Notably, Re(vii) complex methylrheniumtrioxide (MTO)^23,40,41^ and later on Re(v) complexes^42–47^ demonstrated perchlorate reduction under milder conditions, though practical application remains limited by the need for organic reductants. In this study, we introduce a new rhenium(vi) complex (1) that enables controlled perchlorate reduction under comparatively mild conditions. Through integrated spectroscopic, electrochemical, and reactivity analyses, we uncover a distinct oxygen atom transfer pathway at rhenium, advancing molecular-level understanding of Cl–O bond activation. This work not only expands the chemistry of rhenium oxo species but also provides a promising blueprint for designing future catalytic systems for oxyanion remediation.

The unique structure and electronic properties of complex 1 present a new opportunity for exploring its role in OAT catalysis and redox transformations. The stability and efficient formation of this species highlight its potential applications in selective catalytic processes, particularly in oxyanion reduction chemistry.^48^ Although the formation and reactivity of Re μ-oxido complexes of this type are not yet fully understood, the present study provides new insight into their assembly.^49^

## Results and discussion

**Scheme 1:**
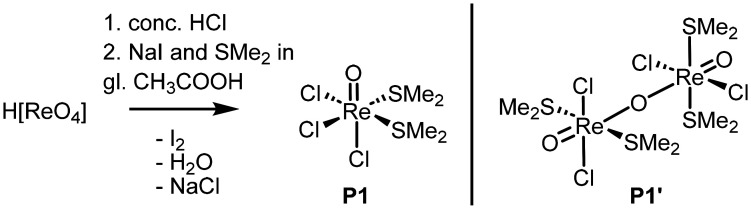
Previously published synthesis of precursor P1 and its hydrolyzed product P1′.

Initially, the synthesis of complex [ReOCl(L1)_2_] was targeted, starting from precursor [ReOCl_3_(OPPh_3_)(SMe_2_)].^50^ In an attempt to simplify the sometimes tedious purification of the complex from side-product OPPh_3_, the phosphine-free precursor [ReOCl_3_(SMe_2_)_2_] (P1) was employed. Precursor complex P1 was prepared with slight modifications to the reported procedure. Starting from HReO_4_, P1 was obtained by reaction in a mixture of concentrated HCl and glacial acetic acid in the presence of KI and dimethyl sulfide (SMe_2_) (Scheme 1).

In contrast to the published procedure of I_2_ removal by vacuum sublimation, we found repeated washing with Et_2_O until the ether remains clear to be more effective, as this procedure yields iodine-free P1 (as evidenced by elemental analysis) as a dark-green product. The obtained green powder is stable in the solid state for several days under ambient conditions, as confirmed by IR spectroscopic comparison. In contrast, precursor P1 shows pronounced solution sensitivity. P1 displays only limited solubility in polar solvents and undergoes partial decomposition upon dissolution. Crystallization attempts of P1 from CH_3_CN afforded, at first the μ-oxido-bridged complex {[ReOCl_2_(SMe_2_)]}_2_(µ-O) (P1′), which forms *via* hydrolysis with trace amounts of water. Only from pre-dried CH_3_CN under inert atmosphere, P1 could be crystallized. The previously unpublished solid-state structures of both P1 and P1′, together with additional characterization data, are provided in the Experimental part. In the solid state, P1 adopts the *fac*-isomer and *cis*-oriented SMe_2_ ligands (Fig. S9). In the ^1^H NMR spectrum of P1 (CD_2_Cl_2_), a single peak at 2.91 ppm (CDCl_3_) is visible for the four SMe_2_ groups (Fig. S1), indicating free rotation around the Re–S bond, thus rendering all four Me groups equivalent. The Re=O stretching vibration is observed at 984 cm^−1^ with relatively low intensity, consistent with the presence of a terminal oxido ligand. The reduced intensity reflects the electronic influence of the *cis*-oriented SMe_2_ ligands, which can partially delocalize electron density onto the Re center and decrease the dipole change associated with the Re=O stretch.^51,52^

When P1 was reacted with two equivalents of HL1, instead of the typical green color of oxidorhenium(v) complexes, an intensely purple colored product was obtained. After several attempts to grow single crystals, X-ray diffraction analysis provided the unexpected structure of 1, revealing ‘deep purple’ to be a µ-oxido bridged di-nuclear rhenium(vi) complex (Fig. 4) rather than the anticipated monomeric oxidorhenium(v) species.

**Scheme 2:**
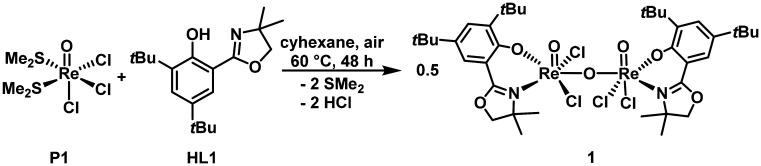
Synthesis of complex 1.

With this information in hand, the synthesis was optimized, giving the best results in cyclohexane at 60 °C over 48 h under ambient conditions, with atmospheric oxygen proving to be the oxidant (*vide infra*). Cyclohexane proved to be the most suitable solvent, yielding complex 1 in over 90% yield under optimized conditions. The choice of cyclohexane as solvent is highly counterintuitive, as both HL1 and P1 show little to no solubility. However, using other solvents like dichloromethane, methanol, ethanol, chloroform or even tetrachloromethane yielded either no reaction or significantly lower product yields. Purification was achieved by crystallization from a 1 : 1 mixture (vol%) of cyclohexane and dichloromethane. To determine the source of the oxygen, the synthesis was performed under inert conditions. In this case, only untraceable mixtures and unreacted starting material were obtained (Scheme 2).

Spectroscopic characterization *via*^1^H NMR spectroscopy showed the expected shifts in the spectrum as well and the appearance of characteristic doublets of the oxazoline methylene moiety (Fig. S3), which usually confirm the coordination to the metal center. Rather surprisingly though, NMR spectroscopy also confirmed the clean, stereoselective formation of only the *cis*/*trans* isomer of 1, with respect to the chlorido ligands on each rhenium center. No signals for the *cis*/*cis*- or *trans*/*trans*-chlorido isomers were observed. Notably, the formation of such a µ-oxido bridged structure is observed exclusively with the *tert*-butyl-substituted ligand HL1. In contrast, other ligands from the HdmozR family (R = H, OMe, NO_2_, Cl) only form mono-nuclear Re(v) analogues.^22,44^

The deep purple color of complex 1 reflects its solid-state structure and associated electronic features. In contrast to mononuclear high-valent Re(v) species, which are typically green, the intense purple color arises from charge-transfer (CT) transitions, as previously observed in related rhenium complexes.^49^ Consistently, UV–Vis spectroscopy revealed a strong absorption band between 480 and 700 nm, with a molar extinction coefficient of *ε* = 5.8 × 10^3^ L mol^−1^ cm^−1^ at 575 nm, characteristic of CT bands in µ-oxido-bridged binuclear complexes.^53,54^

### Cyclovoltammetry

**Fig. 2:**
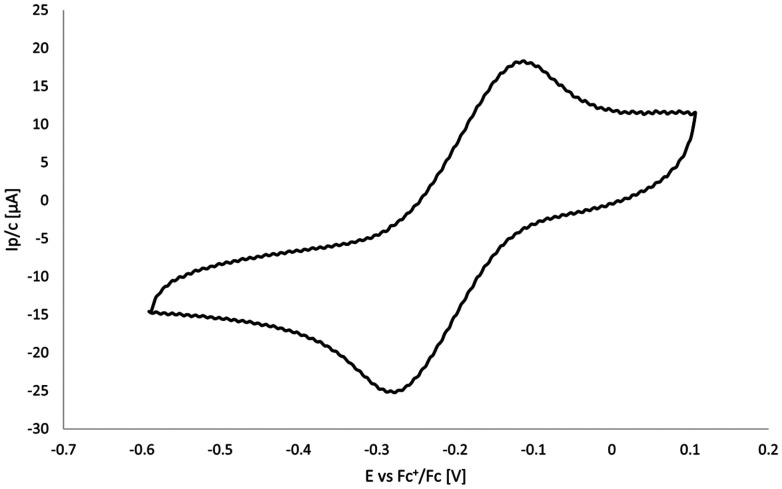
CV of complex 1 in dichloromethane.

The electrochemical properties of complex 1 were investigated by cyclic voltammetry within the potential range −0.5 to +0.2 V in dichloromethane. Anodic scanning revealed an oxidation event at a peak potential of −112 mV with a peak current of 24.8 µA, attributable to the Re(vi)/Re(vii) couple. The relatively low oxidation potential supports this assignment. The corresponding cathodic process was observed at −286 mV with a peak current of 25.2 µA. The near equivalence of anodic and cathodic peak currents (Δ*I* = 0.4 µA) is consistent with a reversible one-electron redox process (Fig. 2).

With an *E*_1/2_ centered at −190 mV, the oxidation potential indicates that 1 is thermodynamically capable of engaging in oxygen atom transfer (OAT) processes. The peak-to-peak separation of 174 mV, however, exceeds the theoretical value for a Nernstian one-electron process, indicating quasi-reversible electron transfer. On this basis, 1 can be classified as a Robin-Day class III-B mixed-valence complex.^55^

This relatively negative oxidation potential indicates that the rhenium center in 1 can be readily oxidized, providing a favorable driving force for oxygen atom transfer. Compared to typical reference compounds such as ferrocene, which require more positive potentials for oxidation, 1 can undergo electron transfer more easily, facilitating the activation and transfer of oxygen atoms.^56^ The quasi-reversible nature of the process, as indicated by the peak-to-peak separation, suggests that some structural reorganization accompanies electron transfer, but the overall thermodynamics remain highly favorable for OAT.^57,58^

### Cleavage of the µ-oxido bridge with aryl phosphines

**Scheme 3:**
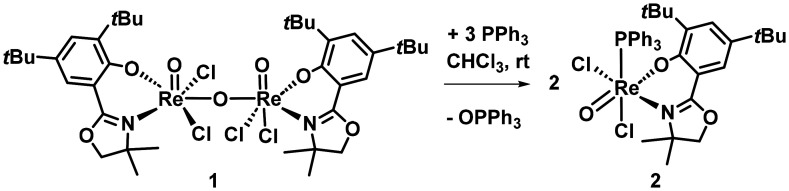
Synthesis of oxidorhenium(v) complex 2.

To investigate the potential cleavage of the µ-oxido bridge in complex 1, as has often been demonstrated in related µ-oxido bridged Mo complexes (*vide infra*), reactions with various phosphines were performed. To assess the influence of oxygen, the reactions were carried out under both inert and ambient conditions, with no observable change in the reaction outcome. However, when triphenylphosphine (PPh_3_) was added to a solution of 1 in chloroform or dibromomethane (Scheme 3), a color change from deep purple to green was observed over several hours, indicating the formation of the new oxidorhenium(v) complex [ReOCl_2_(L1)(PPh_3_)] (2). This transformation was confirmed by ^1^H and ^31^P NMR spectroscopy, which showed distinct signals for the newly formed species and the corresponding phosphine oxide by-product (Fig. S6 and S7). The respective solid state structure of 2 (Fig. 5) was further confirmed by single crystal X-ray diffraction analysis.

Reactions with phosphines bearing alkyl or cycloalkyl substituents, such as trimethylphosphine, tricyclohexylphosphine or tributylphosphine, initially also led to bridge cleavage, as indicated by intermediate color changes, *in situ*-IR-spectroscopy and NMR observations. However, the resulting rhenium(v) complexes were unstable and rapidly decomposed under N_2_, thus preventing their isolation. Based on the unusually shifted signals in the ^1^H NMR spectra, we assume a further reduction, resulting in paramagnetic Re(iii) complexes is occurring with those phosphines. The electron-donating inductive effect (+I) of the alkyl groups could be responsible. In contrast, the aryl phosphine PPh_3_ facilitated bridge cleavage while also stabilizing the resulting Re(v) complex, allowing for successful isolation and characterization.

**Scheme 4:**
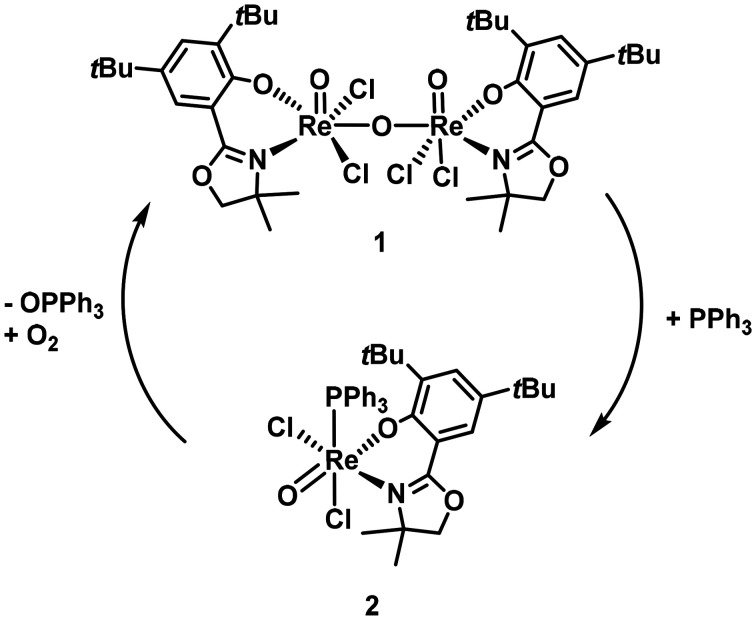
Formation of complex 2 and re-formation of complex 1.

Moreover, successful reformation of complex 1 from 2 was demonstrated by heating the green complex 2 in cyclohexane under air to 60 °C for 48 h. This resulted in the reappearance of the characteristic deep purple colour, and subsequent spectroscopic analysis confirmed the regeneration of complex 1 (Scheme 4). These observations suggest a reversible coordination equilibrium dependent on phosphine ligand properties, highlighting the role of aryl phosphines in modulating the stability of rhenium µ-oxido complexes.

### Isolation of decomposed complex 3 and intermediate product 4

**Fig. 3:**
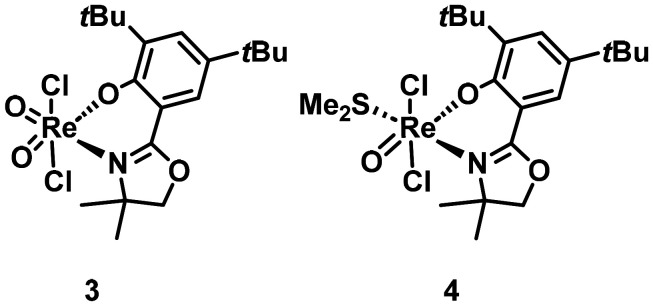
Structure of the Re(vii) complex 3 and the Re(v) complex 4.

From a failed attempt to crystallize 1, the mono-nuclear dioxidorhenium(vii) complex [ReO_2_Cl_2_(L1)] (3, Fig. 3), with the oxido groups *cis* to each other, was obtained. The solid state structure of 3 was confirmed by X-ray diffraction analysis (Fig. 6) and we could also obtain a ^1^H NMR spectrum, although only of low purity (Fig. S8). Complex 3 is a decomposition product of 1*via* disproportionation. In another case, after the synthesis of 1, the intermediate Re(v) product [ReOCl_2_(L1)(SMe_2_)] (4, Fig. 3), could be isolated by crystal picking. Single-crystals of sufficient quality for a XRD measurement could be obtained by recrystallization (Fig. 7). Complex 4 is the initial product formed after coordination of the first L1 ligand, but before the oxidation by air to 1 occurred.

**Fig. 4:**
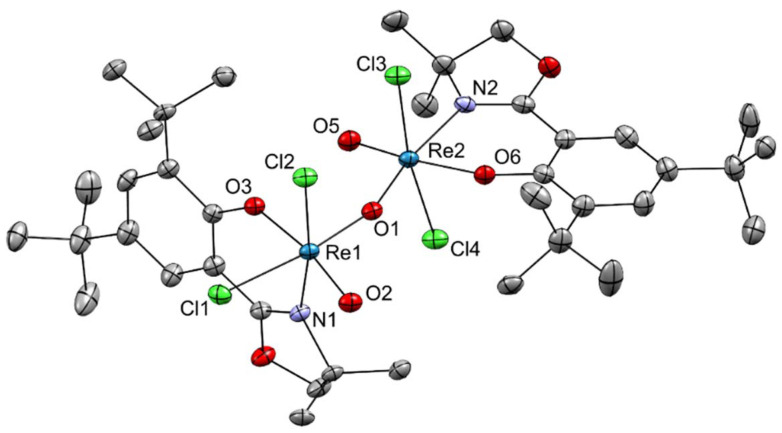
Molecular structure of complex 1. The probability ellipsoids are drawn at 50% probability. The H atoms and a cyclohexane solvent molecule were omitted for clarity. For the disordered *tert*-butyl group, only the positions with the highest occupancies are depicted.

**Fig. 5:**
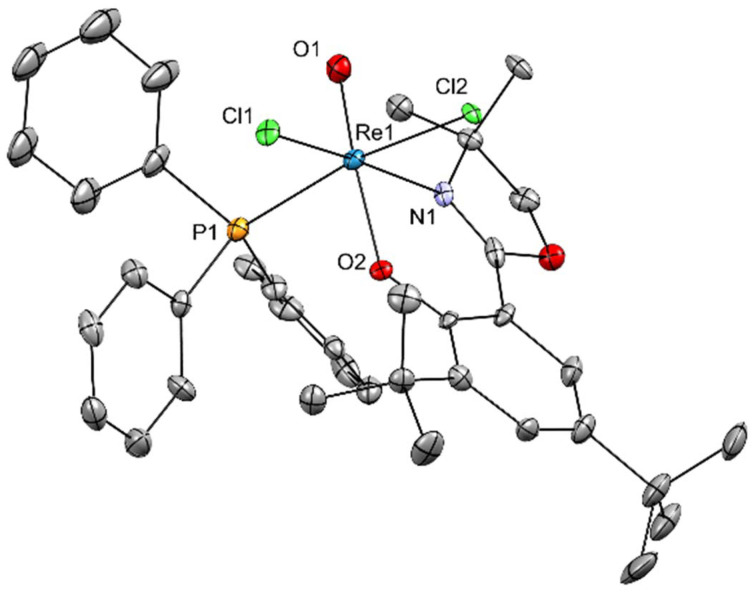
Molecular structure of complex 2. The probability ellipsoids are drawn at 30% probability. The H atoms and a second, independent molecule of 2 in the unit cell were omitted for clarity. For the disordered *tert*-butyl group, only the positions with the highest occupancies are depicted.

**Fig. 6:**
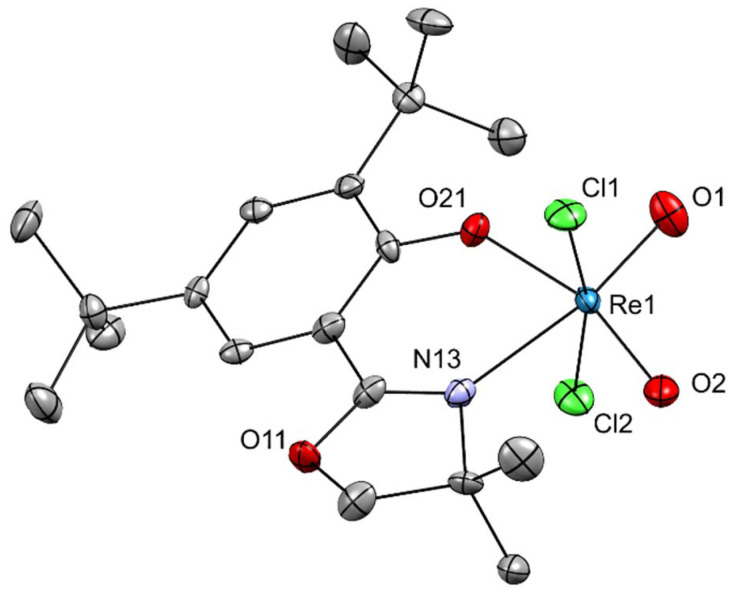
Molecular structure of complex 3. The probability ellipsoids are drawn at 50% probability. The H atoms were omitted for clarity.

**Fig. 7:**
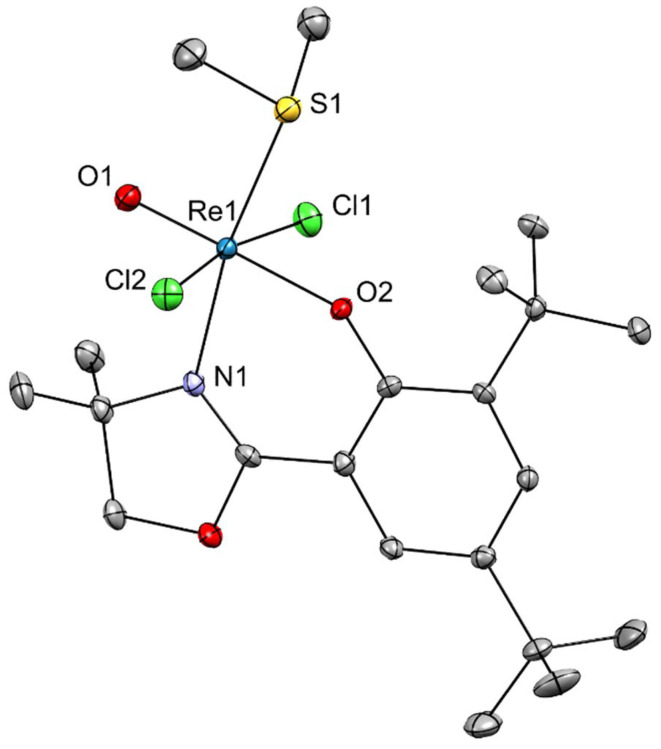
Molecular structure of complex 4. The probability ellipsoids are drawn at 50% probability. The H atoms were omitted for clarity.

The isolation of complexes 3 and 4 offers valuable insight into the possible pathways underlying perchlorate reduction and the formation of 1. In the case of complex 3, the isolation of the Re(vii) species indicates that 1 does undergo disproportionation in solution. The corresponding Re(v) fragment, {ReOCl_2_(L1)}, was not observed, likely due to its coordinative unsaturation. This disproportionation could plausibly represent the initiation step of pre-catalyst 1 in the perchlorate reduction cycle (*vide infra*), with {ReOCl_2_(L1)} serving as the active catalytic species. At present, it remains speculative whether Re(vii) complex 3 itself can participate directly in catalysis. While literature reports suggest that dioxorhenium(vii) and perrhenate complexes are generally inactive in oxygen atom transfer (OAT) reactions, it is conceivable that 3 could engage with SMe_2_ to generate the catalytically competent Re(v) species. Targeted synthesis of 3 to probe this possibility has not yet been successful.

### Solid state structures

Single crystals suitable for X-ray diffraction analysis of the μ-oxido bridged complex {[ReOCl_2_(L1)]}_2_(μ-O) (1) were obtained as dark-purple plate-shaped crystals by slow diffusion of cyclohexane into a dichloromethane solution of the complex. Crystallographic data is summarized in Table S1, and selected bond lengths and angles are listed in Tables 1 and 2. In 1, two Re(vi) centers are connected through a bent μ-oxido bridge (∠(Re1–O1–Re2) = 157.91(19)°), each adopting a distorted octahedral coordination environment. Each rhenium atom is coordinated by two chlorido ligands, one terminal oxido ligand and one bidentate N,O-donor ligand moiety of L1. The overall coordination sphere is typical for bis(oxido)rhenium(vi) complexes.^44,59^ The Re–O(μ) bond lengths (*d*(Re1–O1) = 1.869(3) Å, *d*(Re2–O1) = 1.845(3) Å) fall within the expected range for Re(vi)–O bridging distances and are comparable to previously reported μ-oxido bridged rhenium(vi) complexes.^60^ The terminal Re=O bonds (*d*(Re1–O2) = 1.684(3) Å, *d*(Re2–O5) = 1.688(3) Å) are significantly shorter, confirming their multiple-bond character.^61^ Both Re centers exhibit a *cis*-arrangement of the terminal oxido ligands with respect to the bridging oxido ligand, while the two terminal oxido groups are oriented *trans* to each other across the Re–O–Re axis (∠(O2–Re1–Re2–O5) = 159.15(17)°). This geometry contrasts with the typical *cis* configuration of terminal oxido ligands found in analogous Re(v) oxido dimers. The O donors of the N,O-chelating ligands occupy positions *trans* to the terminal oxido ligands (*d*(Re1–O3) = 1.933(3) Å, *d*(Re2–O6) = 1.922(3) Å). Interestingly, the coordination environment around each rhenium center is asymmetric with respect to the nitrogen donors: one nitrogen atom is located *trans* to a chlorido ligand (*d*(Re1–N1) = 2.113(4) Å, ∠(O1–Re1–N1) = 92.10(14)°), whereas the other is positioned *trans* to the bridging oxido atom (*d*(Re2–N2) = 2.143(4) Å, ∠(O1–Re2–N2) = 170.49(14)°). Selected Re–Cl bond distances (*d*(Re1–Cl1) = 2.3828(11) Å, *d*(Re1–Cl2) = 2.3192(10) Å; *d*(Re2–Cl3) = 2.3445(12) Å, *d*(Re2–Cl4) = 2.3793(11) Å) lie within the range observed for related Re(vi) species.^61^ The coordination geometry is further confirmed by typical bond angles, such as ∠(Cl2–Re1–Cl1) = 89.13(4)° and ∠(Cl3–Re2–Cl4) = 171.38(5)°, indicating a pronounced distortion from ideal octahedral symmetry. Overall, compound 1 represents a rare example of a μ-oxido bridged dioxidorhenium(vi) species featuring bidentate N,O-ligands, exhibiting characteristic Re–O distances and a markedly asymmetric coordination environment around the two Re centers.

**Table 1:** Selected bond lengths (Å) of complexes 1, 2, 3, and 4

Bond [Å]	1 (μ-O)	2 (PPh_3_)	3 (Re(vii))	4 (SMe_2_)
Re=O (terminal)	1.845–1.869	1.681(7)	1.713–1.716	1.691(2)
Re–O (phenolate)	1.688(3)	1.963(6)	1.947(4)	1.929(2)
Re–Cl	2.319–2.387	2.366–2.438	2.328–2.363	2.396–2.415
Re–N (oxazoline)	2.113–2.143	2.160(9)	2.237(5)	2.128(2)
Re–P/S	—	2.490(3)	—	2.4128(8)

**Table 2:** Selected bond angles (°) of complexes 1, 2, 3, and 4

Angle [°]	1 (μ-O)	2 (PPh_3_)	3 (Re(vii))	4 (SMe_2_)
O–Re–O	157.91(19)	171.4(4)	105.1(2)	175.86(10)
Cl–Re–Cl	89–171	88.56(9)	167.46(5)	170.48(3)

The single yellow plate-shaped crystals of [ReOCl_2_(L1)(PPh_3_)] (2) were obtained by slow diffusion of *n*-hexane into a dichloromethane solution. The complex crystallized in the non-centrosymmetric space group *P*2_1_ with two independent molecules in the asymmetric unit. Crystallographic data are summarized in Table S2 and selected bond lengths and angles are listed in Tables 1 and 2. Complex 2 features a distorted octahedral coordination environment around the rhenium center (Fig. 5). The coordination sphere consists of one terminal oxido ligand *trans* to the phenolato oxygen of the chelating oxazolinato ligand (*d*(Re1–O1) = 1.681(7) Å, *d*(Re1–O2) = 1.963(6) Å; ∠(O1–Re1–O2) = 171.4(4)°). Two chlorido ligands occupy *cis* positions (*d*(Re1–Cl1) = 2.366(3) Å, *d*(Re1–Cl2) = 2.438(2) Å; ∠(Cl1–Re1–Cl2) = 88.56(9)°), while the oxazoline nitrogen lies *trans* to chlorido ligand Cl1 (*d*(Re1–N1) = 2.160(9) Å, ∠(N1–Re1–Cl1) = 171.5(3)°). The triphenylphosphine ligand completes the octahedral coordination *trans* to the second chlorido ligand Cl2 (*d*(Re1–P1) = 2.490(3) Å, ∠(Cl2–Re1–P1) = 170.45(10)°). The Re=O bond distance (1.68 Å) is in the expected range for oxidorhenium(v) species and comparable to that in related [ReOCl_2_(L)] complexes.^44^ All remaining bond lengths and angles fall within the expected ranges (Tables S9 and S10). Structurally, 2 closely resembles previously reported oxidorhenium(v) complexes containing mixed donor ligands such as phosphines and N,O-chelates, including those described by Schachner *et al.*^44^ The *cis* arrangement of the chlorido ligands and the *trans* disposition of the oxido and phenolato donors are characteristic for distorted octahedral {Re(v)OCl_2_} cores. In comparison to the μ-oxido bridged Re(vi) complex 1, the Re=O bond in 2 is slightly shorter, consistent with the higher multiple-bond character and the absence of a bridging oxido ligand.

For [ReO_2_Cl_2_(L1)] (3) single crystals of high enough quality were obtained by slow diffusion of *n*-heptane into a dichloromethane solution of 1. The crystal structure is shown in Fig. 6 and selected bond lengths and angles are listed in Tables 1 and 2. The rhenium center exhibits a distorted octahedral coordination geometry, formed by two terminal *cis*-oxido ligands, two chlorido ligands in *trans* position (*d*(Re1–Cl1) = 2.3280(13) Å, *d*(Re1–Cl2) = 2.3631(13) Å; ∠(Cl1–Re1–Cl2) = 167.46(5)°) and the bidentate N,O-chelating ligand L1. The short Re=O distances (*d*(Re1–O1) = 1.713(4) Å, *d*(Re1–O2) = 1.716(4) Å) confirm their multiple-bond character, while the Re–O(phenolate) and Re–N(oxazoline) bonds (*d*(Re1–O21) = 1.947(4) Å, *d*(Re1–N13) = 2.237(5) Å) fall within the expected range for such Re(vii) complexes.^44^

The two terminal oxido ligands adopt a distorted *cis* conformation (∠(O1–Re1–O2) = 105.1(2)°) and are nearly *trans* to the donor atoms of the chelating ligand (∠(O1–Re1–N13) = 169.48(19)°, ∠(O2–Re1–O21) = 162.44(18)°). The overall coordination sphere therefore reflects the typical moderate distortions from ideal octahedral symmetry. When compared to the μ-oxido bridged Re(vi) complex [ReOCl_2_(dmoz(*t*Bu_2_))]_2_(μ-O) (1), complex 3 displays noticeably shorter Re=O bond lengths and more linear Cl–Re–Cl axes. In contrast to 1, which features a *cis* arrangement of terminal oxido ligands relative to a bridging μ-oxido group, 3 contains two equivalent terminal oxido ligands coordinated in a compact *cis* geometry around a single Re(vii) center.

Yellow plate-shaped single crystals of [ReOCl_2_(L1)(SMe_2_)] (4) were obtained from a dichloroethane solution of 1 by slow evaporation and analyzed by X-ray diffraction. The complex features a distorted octahedral coordination around the rhenium(v) center. The coordination sphere consists of a terminal oxido ligand (*d*(Re–O1) = 1.691(2) Å) *trans* to the phenolato oxygen (*d*(Re–O2) = 1.929(2) Å; ∠(O1–Re–O2) = 175.86(10)°), two chlorido ligands in *trans* positions (*d*(Re–Cl1) = 2.4149(8), *d*(Re–Cl2) = 2.3962(7) Å; ∠(Cl1–Re–Cl2) = 170.48(3)°), and the oxazoline nitrogen (*d*(Re–N1) = 2.128(2) Å) *trans* to the dimethylsulfide ligand (*d*(Re–S1) = 2.4128(8) Å). All bond lengths and angles are consistent with a low-spin Re(v) center in a distorted octahedral geometry. Compared to the phosphine complex 2, the softer SMe_2_ donor gives a slightly longer Re–S bond and marginally shorter Re–O(phenolate) distance, indicating stronger π-donation from sulfur. In contrast to the μ-oxido Re(vi) complex 1 and the dioxidorhenium(vii) complex 3, complex 4 retains a mononuclear Re(v) core with a single terminal oxido ligand. The *trans*-[O=Re–O(phenolate)] arrangement is preserved across all oxidation states, illustrating the structural strength of the oxazolinato–phenolato coordination in stabilizing the rhenium-oxido species.

### Computational chemistry

**Fig. 8:**
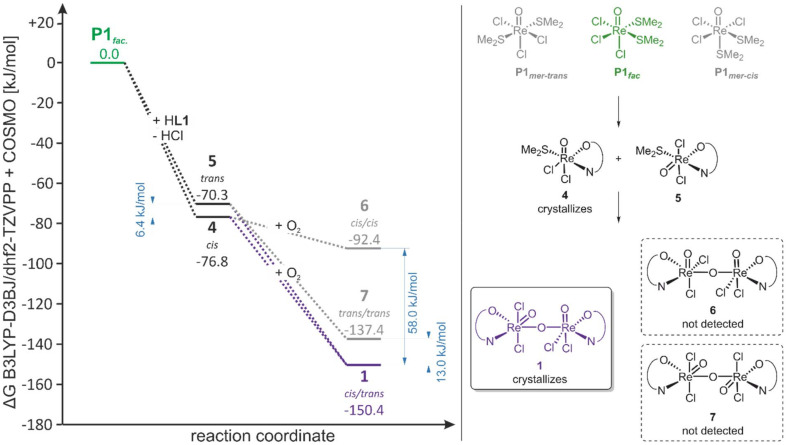
Free energy profile of the formation of complex 1 referenced to the *fac*-chlorido isomer of P1. Energies of 1, 6 and 7 are given per rhenium atom to remain consistent with the reaction equation.

To shed light on the factors governing the stereoselective formation of complex 1, DFT calculations were carried out at the B3LYP-D3BJ/dhf-TZVPP//B3LYP-D3BJ/dhf-SVP level of theory and employing the COSMO solvation model for cyclohexane (see section “Computational details” in the SI). The obtained free energy profile (Fig. 8) highlights the key steps of the reaction pathway leading from precursor [ReOCl_3_(SMe_2_)_2_] (P1) to complex {[ReOCl_2_(L1)]}_2_(μ-O) (1).^62–80^

Precursor P1 could in principle exist in three different configurational isomers, the *fac*-chlorido structure P1_*fac*_ and two *mer*-chlorido isomers P1_*mer-trans*_ and P1_*mer-cis*_ (with the DMS *trans* and *cis* to each other, respectively). Here, P1_*fac*_ was taken as a reference, since this configuration is consistent with its determined solid state structure (Fig. S9) and also fits the fact that it was determined to exhibit the overall lowest energy (with P1_*mer-trans*_ and P1_*mer-cis*_ being 3.2 and 12.4 kJ mol^−1^ less stable, see SI).

Formation of both the isolated *cis*-dichlorido isomer [ReOCl_2_(L1)(SMe_2_)] (4) and calculated *trans*-dichlorido isomer 5 of [ReOCl_2_(L1)SMe_2_] from P1_*fac*_ is exergonic (−76.8 and −70.3 kJ mol^−1^, respectively), which adds further support to their role as reaction intermediates. The lower energy of the *cis*-isomer 4 is consistent with its isolation in experiment. As the Gibbs free energies of 4 and 5 differ by only 6.4 kJ mol^−1^, both building-blocks required for the formation 1 are indicated to co-exist in solution, with the calculated energy difference translating to a ratio of complexes 4 : 5 of approximately 13 : 1 (as obtained from a Boltzmann distribution).

From complexes 4 and 5, three potential binuclear rhenium complexes could be formed, the crystallized *cis*/*trans*-form 1 and the hypothetical *all cis* and *all trans* (with respect to the four chlorido ligands) isomers 6 and 7. For these complexes, energies are given per rhenium center to remain consistent with the reaction equation. Formation of all possible dinuclear complexes from their mononuclear precursors is exergonic (Fig. 8). Upon comparing the energies of the crystallized rhenium-complex 1, 6 and 7, it becomes evident that 1 is 58.0 kJ mol^−1^ and 13.0 kJ mol^−1^ more stable, respectively. This provides a thermodynamic argument for the stereoselective formation of complex 1.

As in equilibrium, there exists a significantly larger amount of *cis*-4 than of *trans*-5, formation of the *all cis* dinuclear complex 6 is statistically the most likely. However, due to the low energy gain of the reaction from 4 to 6 (−15.6 kJ mol^−1^) and considering the elevated reaction temperatures, the formation of 6 is postulated to be reversible, rendering species 6 unstable. Contrarily, formation of complexes 1 or 7 can be considered irreversible (ΔΔ*G* = 60.7 and 73.6 kJ mol^−1^), but, due to the low amount of *trans*-5 present in solution, formation of the *all trans* complex 7 is statistically strongly unfavored, ultimately leading to stereoselective formation of “deep purple” complex 1, being the final reaction product. It should be noted, that if considering all reactions to be reversible would lead to the same picture, as 1 represents the global minimum possible isomers.

### Catalytic studies

Catalytic oxygen atom transfer (OAT) reactivity was evaluated for complex {[ReOCl_2_(L1)]}_2_(μ-O) (1) in comparison to isoelectronic µ-oxido bridged molybdenum(v) analogues, that formally share the same d^0^ to d^2^ redox couples, Re(vii)/Re(v) *versus* Mo(vi)/Mo(iv).^81^

**Scheme 5:**
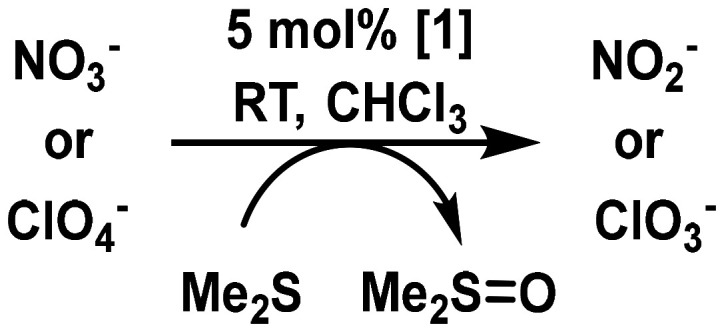
General scheme of first catalytic step of oxyanion reduction.

**Fig. 9:**
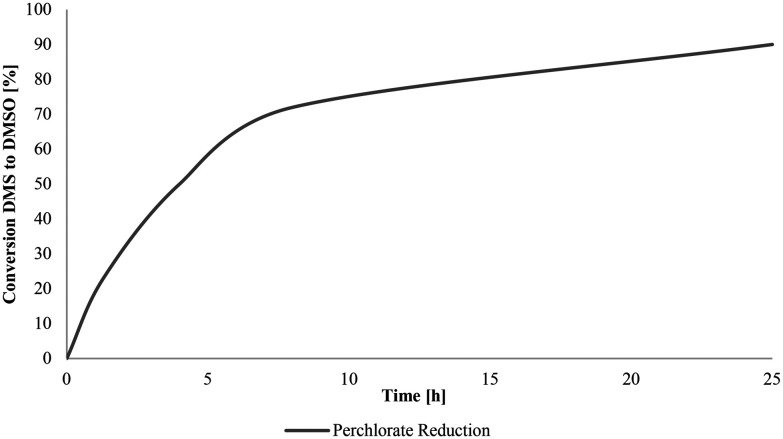
Time-conversion plot of ClO_4_^−^ reduction.

Scheme 5 illustrates the first step of the targeted catalytic oxyanion reduction for nitrate and perchlorate. Both occur *via* a 2-electron oxygen atom transfer (OAT). In contrast to nitrate, for perchlorate, also the next three steps occur *via* 2-electron OAT steps to finally yield chloride. For this purpose, 5 mol% of 1 in CDCl_3_, 1 equivalent NBu_4_ClO_4_ and 4 equivalents of SMe_2_ (DMS) as sacrificial oxygen acceptor were used. To determine the catalytic activity, the conversion from DMS to DMSO was tracked *via*^1^H NMR spectroscopy. The result for perchlorate is shown in Fig. 9. In case of perchlorate reduction, a 90% conversion was achieved after 25 h, corresponding to a TON of 18 and a TOF of 0.72 h^−1^.

In nitrate reduction however, the reduction of NO_2_^−^ occurs only *via* an 1-electron reduction giving NO and a singly-oxidized Re(vi) species, that is usually not catalytically active anymore.^44,82^ In addition, NO is easily oxidized by air to NO_2_, a strong oxidant that can also oxidize DMS to DMSO, which results in false turnovers. Nevertheless, we were interested to test if complex 1 shows in principle catalytic activity also in nitrate reduction. Using again 5 mol% of 1 in CDCl_3_, NBu_4_NO_3_ and 5 equivalents of DMS, under ambient conditions, we observed a maximum of 45% conversion to DMSO after 8 h, after which, no more DMSO was produced up to 25 h, proving that complex 1 is also an active nitrate reduction catalyst. However, at this moment, we can neither explain the 45% conversion, nor the apparent halting of conversion at 45% after 8 h. If only 1 turnover from NO_3_^−^ to NO_2_^−^ had occurred (due to the deactivation of the catalyst caused by the 1-electreon reduction of NO_2_^−^ to NO), a conversion of only 20% DMS to DMSO would be expected. We can currently not account for the additional 25% DMSO, that we observed. Also it is surprising that if NO was formed, why a full conversion of DMS to DMSO was not happening, based on the aerial oxidation of NO to the strong oxidizer NO_2_, as we had previously observed.^82^ Hence, the diverting reactivity of 1 compared to previously published complexes of type [ReOCl(L)_2_] is subject to further mechanistic investigations in our labs.

While such redox catalysis is rare with µ-oxido bridged Re(vi) complexes like 1, there is a significant body of work employing dioxidomolybdenum(vi) complexes in redox catalysis as biomimetic models of molybdoenzymes. Nordlander *et al.*^81^ reported [MoO_2_(L–O)]^+^ complexes that react with tertiary phosphines in protic media to generate the reduced mono-oxido species [MoO(L–O)(OCH_3_)], which subsequently catalyze OAT reactions with substrates such as dimethyl sulfoxide or nitrate. The overall Mo(vi) → Mo(iv) → Mo(vi) sequence mimics the functional cycle of molybdoenzymes, but catalysis is limited by rapid dimerization of the Mo(v) intermediates yielding μ-oxido bridged Mo(v) species and by the thermodynamic rigidity of the strong Mo–O bond. As a result, only stoichiometric or a few-turnover OAT processes are observed.^24^

The high activity of rhenium catalysts in perchlorate reduction probably stems from the more labile Re–O bonds, which are less covalent than Mo–O bonds, allowing for easier oxygen uptake and release. The relativistic stabilization of the 5d orbitals lowers the activation barrier for Re–O bond cleavage and enables flexible five-coordinate geometries during the oxygen atom transfer process.^44,58^

## Conclusion

In summary, we report the successful synthesis, characterization, and reactivity of a novel µ-oxido bridged di-nuclear rhenium(vi) complex 1, achieved through an optimized synthesis using cyclohexane as the solvent and air as the oxidant. The unexpected di-nuclear structure, confirmed by X-ray crystallography, shows special electronic properties, including an intense deep purple coloration resulting in a strong charge-transfer absorption band, distinguishing 1 from previously reported high-valent rhenium species.^22,44^ Experiments conducted under nitrogen show that oxygen from air is required for the formation of 1.

The µ-oxido bridge in complex 1 can be reversible cleaved with triphenylphosphine, yielding the rhenium(v) complex 2. The successful regeneration of complex 1 from these derivatives highlights its stability and suitability for catalytic applications. Additionally, the isolation of a dioxidorhenium(vii) complex 3 and the intermediate product 4 provided mechanistic insights into both the formation and the catalytic mechanism of 1.

Computational studies using DFT revealed the thermodynamic landscape of the synthesis, giving an explanation for the observed *cis*/*trans* stereoselectivity and high yield of complex 1. Catalytic studies demonstrated its efficacy in oxygen atom transfer (OAT) reactions, achieving an almost full (90%) reduction of perchlorate chloride. In nitrate reduction, also catalytic activity is observed, and further investigations are currently underway.

The unique structural and electronic properties of complex 1, combined with its catalytic activity, position it as a promising new candidate for selective redox transformations and oxyanion reduction chemistry.

## Experimental part

All experiments, unless otherwise specified, were conducted using commercially available chemicals and solvents without further purification. Inert atmosphere experiments were carried out under nitrogen (N_2_) using standard Schlenk techniques and a glovebox. Dry solvents were sourced from a Pure Solv® solvent purification system. ^1^H, ^13^C and ^31^P NMR spectra were recorded with a Bruker Avance (300 MHz) instrument. Chemical shifts are given in ppm and are referenced to tetramethylsilane (TMS) (app. = apparent). UV/Vis spectra were recorded on a Varian Cary® 50 UV-Vis spectrophotometer, with all measurements conducted in 1 cm quartz cuvettes using acetonitrile solutions. Temperature control for the cuvette holder was achieved using a cryostat. Kinetic studies to determine rate constants were analyzed using the associated software from Agilent Technologies CaryUV®. Mass spectra were recorded with an Agilent 5973 MSD – direct probe using the EI ionization technique. HR-MS (ESI) measurements were recorded with an Agilent Technologies 6230 TOF LC/MS with ESI or APCI mass spectrometer in positive and negative ion mode, the used solvent was acetonitrile or methanol. Peaks are denoted as cationic or anionic mass peaks, and the unit is the according ions mass/charge ratio. Elemental analyses were carried out using a Heraeus Vario Elementar automatic analyzer at the University of Technology, Graz. Samples were measured in duplicate. Cyclic voltammetry was performed inside a glovebox under a nitrogen atmosphere using a Gamry Instruments Reference® 600 potentiostat. Approximately 1 mM analyte solutions were prepared in dry acetonitrile with 100 mM (Bu_4_N)PF_6_ as the supporting electrolyte. A glassy carbon working electrode, a platinum wire (99.99%) counter electrode, and a silver wire reference electrode (immersed in a solution of 10 mM AgNO_3_ and 100 mM (NBu_4_)PF_6_ in acetonitrile, separated by a Vycor® tip) were used. Measurements were recorded with 10 cycles at scan rates of 50–500 mV s^−1^ and referenced to ferrocene. NMR measurements were conducted on a Bruker Avance® (300 MHz) instrument, with spectra referenced to residual solvent signals. IR spectra were obtained using a Bruker Optics ALPHA® FT-IR spectrometer equipped with an ATR diamond probe head.

### Synthesis of P1 and HL1

Precursor [ReOCl_3_(SMe_2_)_2_] P1 ^83^ and ligand Hdmoz(*t*Bu_2_) (HL1)^44^ where synthesized according to literature. Previously unpublished analytical data as well as the solid state structure of P1 can be found in the SI.

#### Synthesis of {[ReOCl_2_(L1)]}_2_(µ-O) (1)

A 100 ml flask was charged with P1 (1 g, 2.3 mmol), HL1 (0.7 g, 2.3 mmol) and 40 mL of cyclohexane. The suspension was stirred at 60 °C for 48 h. The dark purple product was isolated by crystallization (cyclohexane/DCM) (93%, 1.26 g, 1.07 mmol). ^1^H NMR (300 MHz, CDCl_3_) *δ* 7.76 (d, *J* = 2.5 Hz, 1H, ar. H), 7.75 (d, *J* = 2.5 Hz, 1H, ar. H), 7.62 (app. t, 2H, ar. H), 4.49 (d, *J* = 8.55 Hz, 1H, –C*H*_2_–, oxazoline), 4.47 (d, *J* = 8.33 Hz, 1H, –C*H*_2_–, oxazoline), 4.40 (d, *J* = 8.55 Hz, 1H, –C*H*_2_–, oxazoline), 4.29 (d, *J* = 8.33 Hz, 1H, –C*H*_2_–, oxazoline), 1.99 (s, 3H, –C*H*_3_), 1.73 (s, 3H, –C*H*_3_), 1.68 (s, 3H, –C*H*_3_), 1.60 (s, 3H, –C*H*_3_), 1.51 (s, 9H, *t*Bu), 1.49 (s, 9H, *t*Bu), 1.33 (s, 9H, *t*Bu), 1.32 (s, 9H, *t*Bu). ^13^C NMR (75 MHz, CDCl_3_) *δ* 131.76, 130.47, 124.89, 123.17 (all ar. C), 79.74, 78.45 (–*C*H_2_–, oxazoline) 31.57 (*t*Bu), 31.51 (*t*Bu), 30.03 (–*C*H_3_), 29.80 (–*C*H_3_), 29.78 (*t*Bu), 29.50 (–*C*H_3_), 28.05 (–*C*H_3_) 27.02 (*t*Bu). ATR-IR (cm^−1^): 2957.3 (m), 2867.3 (w) 1548.7 (vs) (*ν* C=N), 1411.0 (w), 1383.3 (m), 1267.1 (m), 1201.3 (m), 964.9 (s) (*ν* Re=O), 864.6 (s), 755.5 (m), 553.3 (m), 457.6 (m); UV-Vis (CH_2_Cl_2_) *λ*_max_, nm (*ε*, dm^3^ mol^−1^ cm^−1^): 575 (5800). Samples for HR-MS (ESI) was submitted but due to low solubility of 1 in methanol and CH_3_CN, no meaningful spectral data could be acquired. Elemental analysis calculated for C_38_H_56_Cl_4_N_2_O_7_Re_2_ (1167.09 g mol^−1^) [%]: C 39.11, H 4.84, N 2.40; found: C 38.05, H 4.56, N 2.13.

#### Synthesis of [ReOCl_2_(L1)(PPh_3_)] (2)

A 100 ml flask was charged with 1 (1 g, 0.9 mmol), 3 equiv. of PPh_3_ (0.67 g, 2.6 mmol) and 40 mL of chloroform. The suspension was stirred at 25 °C for 30 minutes. The green product was isolated by precipitation by adding cyclohexane to the solution. Approximately 0.85 g a greenish, poly-crystalline material of 2 can be isolated by this procedure, however, always contaminated with PPh_3_ and OPPh_3_. ^1^H NMR (300 MHz, CDCl_3_): *δ* 7.65 (d, *J* = 2.5 Hz, 1H, ar. H), 7.57–7.35 (m, 15H, P*Ph*_3_), 7.31 (d, *J* = 2.5 Hz, 1H, ar. H), 4.30 (d, *J* = 8.1 Hz, 1H, –*C*H_2_–, oxazoline), 3.64 (d, *J* = 8.1 Hz, 1H, –*C*H_2_–, oxazoline), 1.67 (s, 3H, –C*H*_3_), 1.31 (s, 9H, *t*Bu), 0.84 (s, 9H, *t*Bu), 0.73 (s, 3H, –C*H*_3_). ^13^C NMR (75 MHz, CDCl_3_) *δ* 137.39, 137.24, 135.12, 135.00, 134.01, 133.76, 128.86, 128.68, 128.59 (all ar. C), 77.93 (–*C*H_2_–, oxazoline), 77.89 (–*C*H_2_–, oxazoline), 31.65 (*t*Bu), 31.47 (*t*Bu), 29.55 (–*C*H_3_), 27.06 (–*C*H_3_). ^31^P NMR (121 MHz, CDCl_3_) *δ* −23.17. EI-MS (*m*/*z*): 807 (M^+^-2CH_3_).

### Synthesis from complex 2 to complex 1

The re-formation of 1 was carried out by stirring a cyclohexane solution of complex 2 under ambient atmosphere at 60 °C overnight. The color of the solution gradually changed from greenish to deep purple. Slow evaporation of the solvent led to the crystallization of complex 1. The obtained product is consistent with analytical data obtained from an authentic sample of complex 1.

## Author contributions

T. A. D.: investigation, data curation, formal analysis, writing – original draft. G. O.: investigation, data curation. P. E. H., A. D. B., A. D., F. B.: formal analysis, writing – original draft. J. A. S.: conceptualization, funding acquisition, supervision, writing – review & editing.

## Conflicts of interest

The authors declare no conflicts of interest.

## Supplementary Material

DT-055-D6DT00039H-s001

DT-055-D6DT00039H-s002

## Data Availability

Details regarding X-ray data collection and DFT calculations as well as NMR data of novel compounds are available within the article and its supplementary information (SI). Metadata is deposited at Zenodo under the https://doi.org/10.5281/zenodo.17864719. Supplementary information is available. See DOI: https://doi.org/10.1039/d6dt00039h. CCDC 1913784 (P1′), 1913785 (P1), 1913788 (3), 2500256 (1), 2500257 (2) and 2500259 (4) contain the supplementary crystallographic data for this paper.^84*a*–*f*^
