# Supplementary material for: A di-nuclear μ-oxido rhenium(vi) complex: from deep purple to catalytic applications
Source: Dalton Trans. 2026 Feb 27;55(11):4547–59. doi: 10.1039/d6dt00039h (PMC12947235; doi:10.1039/d6dt00039h)
Supplement: DT-055-D6DT00039H-s001 [file DT-055-D6DT00039H-s001.pdf]

Supplementary Information (SI) for Dalton Transactions.
This journal is © The Royal Society of Chemistry 2026

Supporting information for

A di-nuclear μ-oxido rhenium(VI) complex: from Deep Purple to catalytic applications

Tobias A. Doliner,[†] Peter E. Hartmann,[†] Gernot Oberwinkler,[†,a], Ferdinand Belaj,[†] Antoine Dupé[†], A. Daniel Boese[†] and Jörg A. Schachner,[†,*]

[†]Institute of Chemistry, University of Graz, Schubertstr. 1, 8010 Graz

[a]current affiliation: Kanzler Verfahrenstechnik GmbH, Ragnitzstr. 35, 8047 Graz.

corresponding author: joerg.schachner@uni-graz.at

Analytical data (previously unpublished) for **P1**: ^{1}H NMR (300 MHz, $CDCl_3$) δ 2.91 (s, 6H); ^{13}C NMR (75 MHz, CDCl3) δ 27.65; ATR-IR (cm^{-1}): 2998.4 (w), 2921.4 (w), 1427.1 (m), 1322.8 (m), 1036.6 (m), 984.6 (m) (ν Re=O), 689.1 (s), 670.3 (s); Elemental analysis calculated for $C_4H_{12}Cl_3OReS_2$ (432.82 g/mol) [%]: C 11.10 H 2.79, S 14.81; found: C 11.65, H 2.92, S 15.02.

NMR data

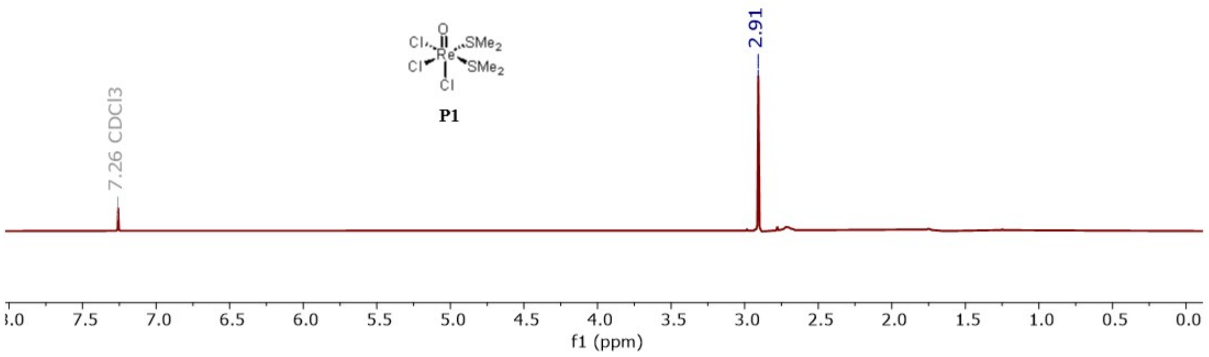

Fig. S1. ^{1}H NMR spectrum of complex **P1**.

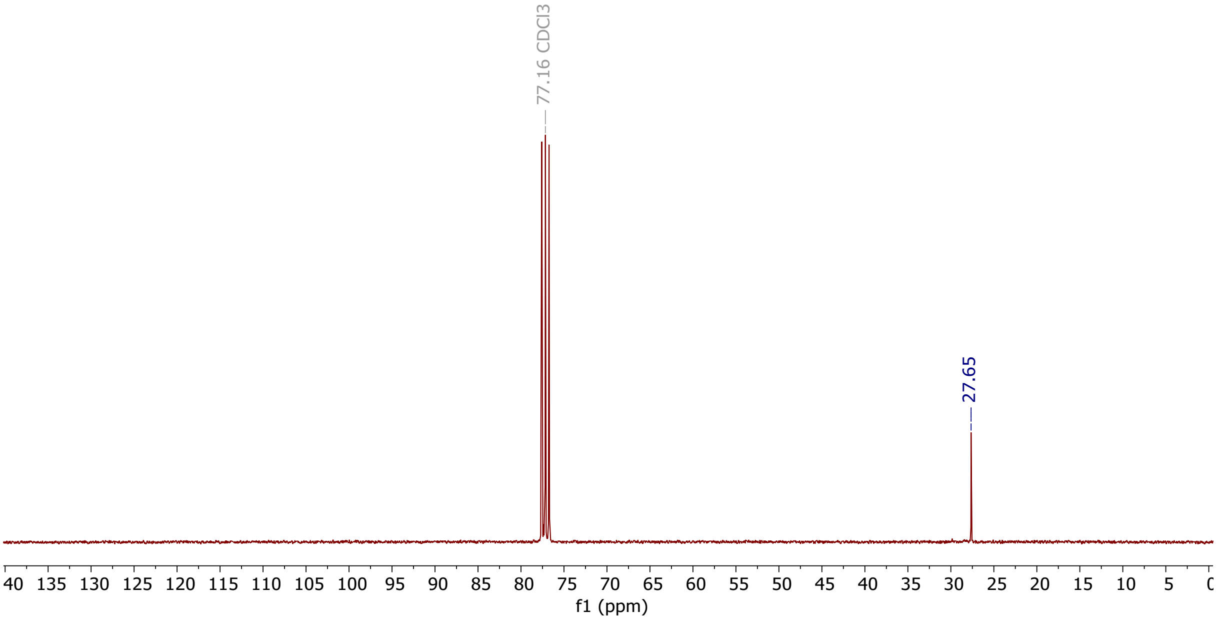

Fig. S2. ^{13}C NMR spectrum of complex **P1**.

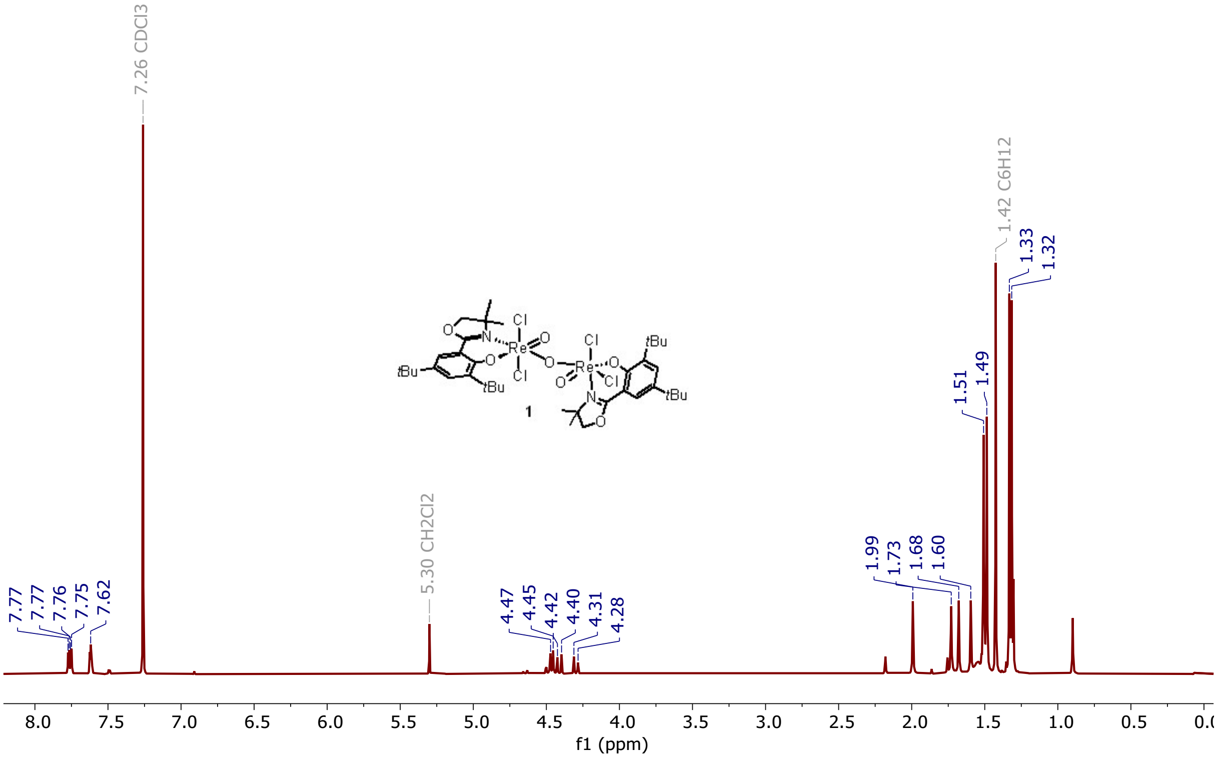

Fig. S3. ^{1}H NMR spectrum of complex **1**

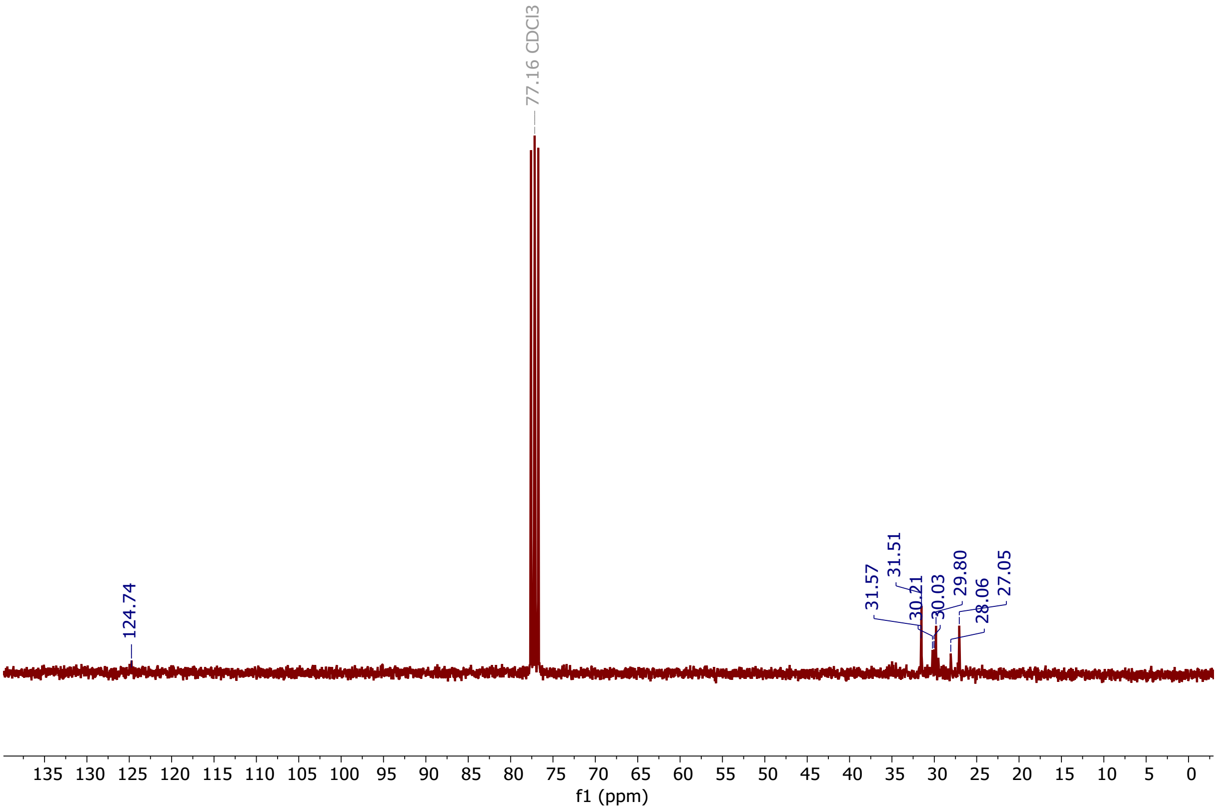

Fig. S4. ^{13}C NMR of complex **1**. Most signals are obscured due to the low solubility of **1**.

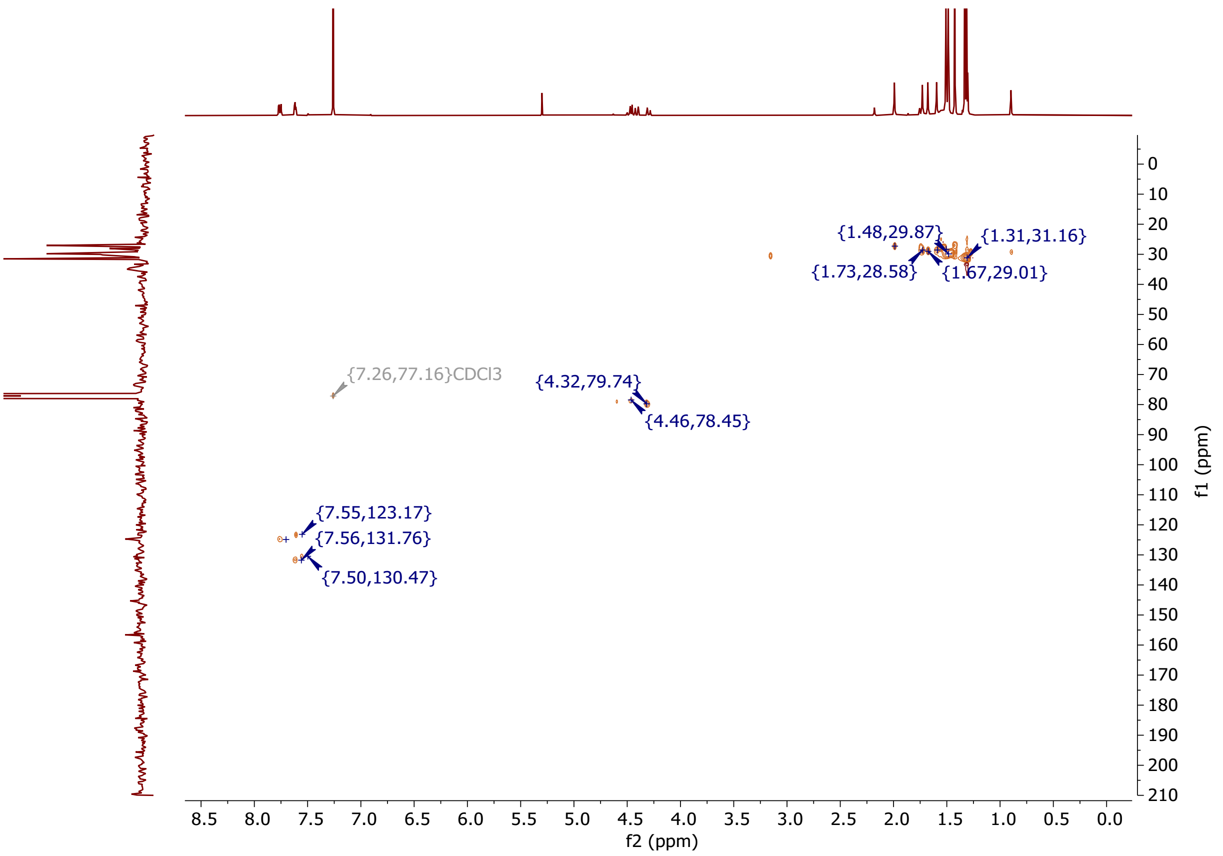

Fig. S5. HSQC spectrum of complex **1**.

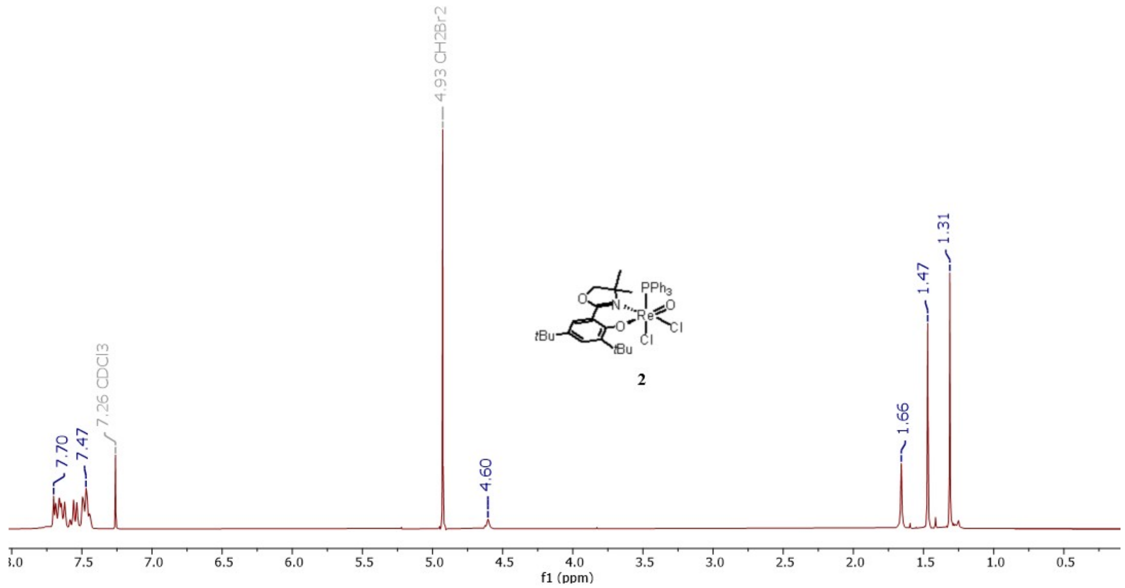

Fig. S6. ^{1}H NMR spectrum of complex **2**.

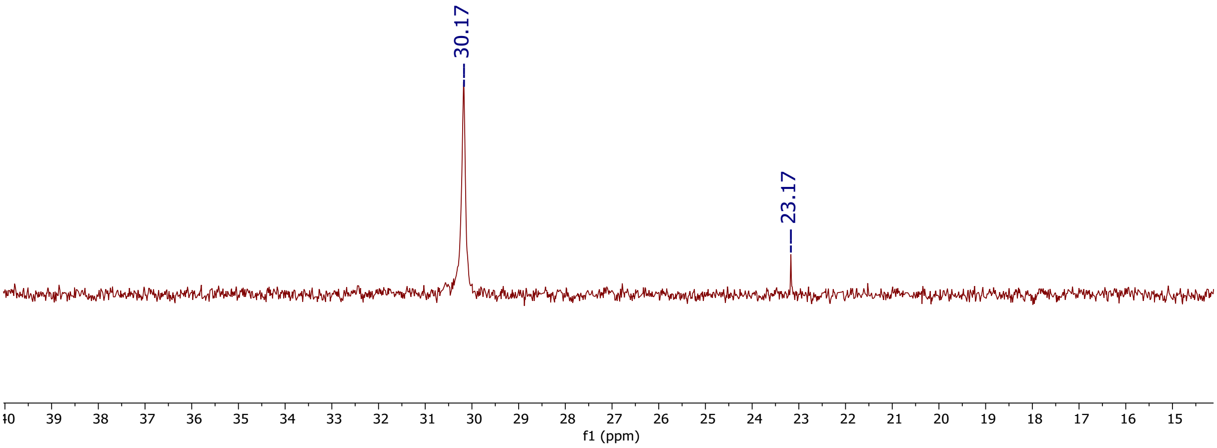

Fig. S7. ^{31}P NMR spectrum of the reaction mixture of complex **1** with 2 equiv. of PPh_3 to give **2** and $OPPh_3$.

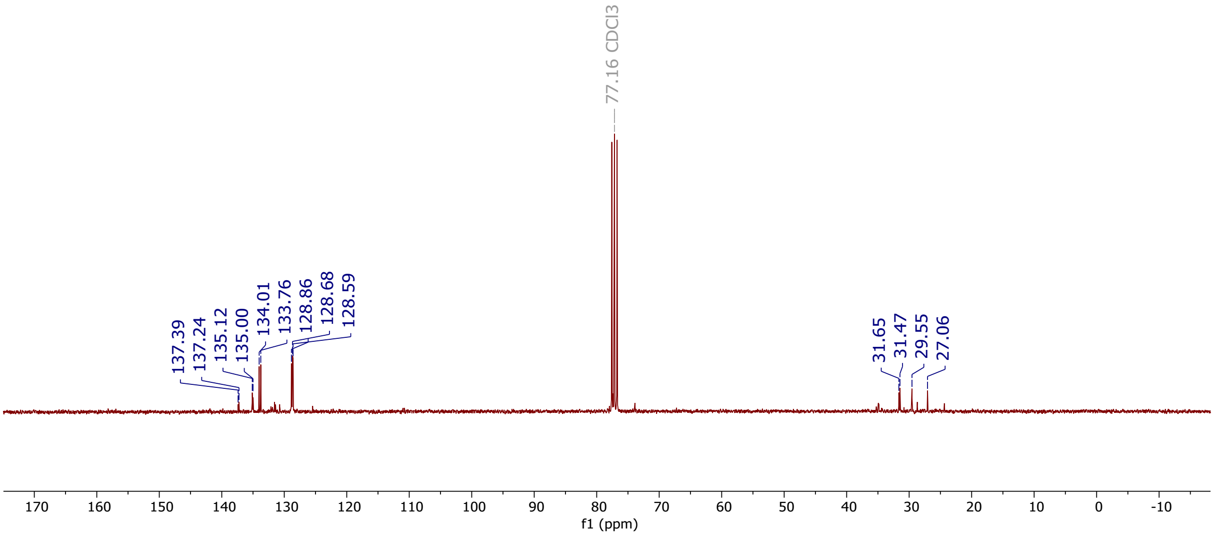

Fig. S8. ^{13}C NMR spectrum of the reaction mixture of complex **1** with 2 equiv. of PPh_3 to give **2** and $OPPh_3$.

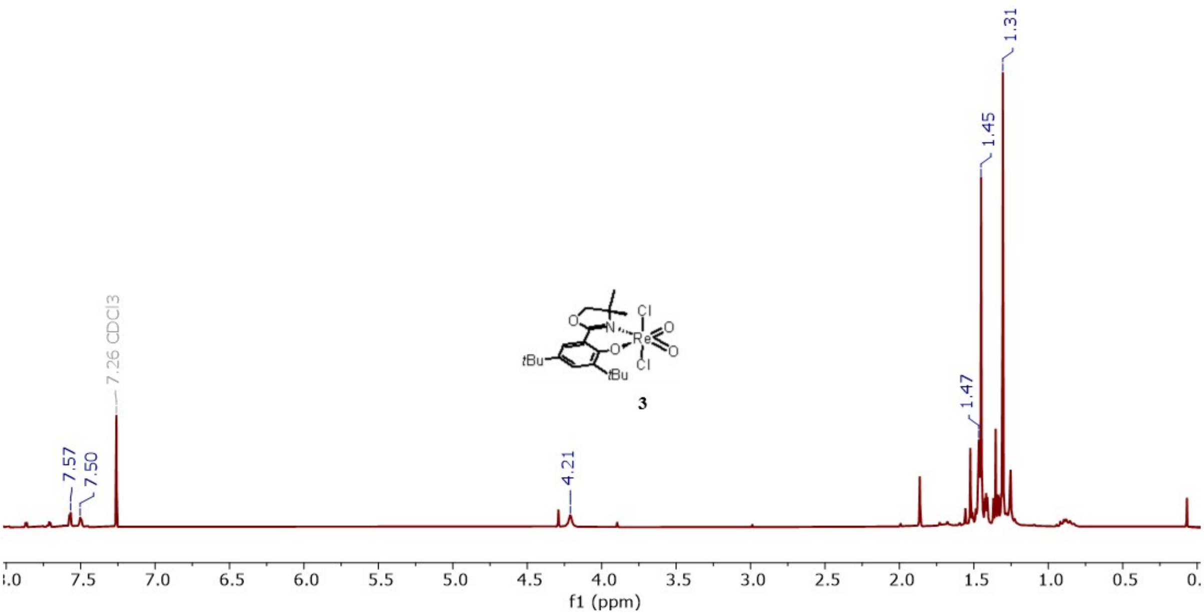

Fig. S9. ^{1}H NMR spectrum of crude material of complex **3**.

X-Ray diffraction analysis

The X-ray data collections for complexes **P1**, **P1′** and **3** were performed with a Bruker AXS SMART APEXII CCD diffractometer at 100 K with Mo Kα radiation (λ= 0.71073 Å) from an Incoatec microfocus sealed tube equipped with a multilayer monochromator. Absorption corrections were made semi-empirically from equivalents. The structures were solved by direct methods (SHELXS-97)[1] and refined by full matrix least-squares techniques against F^2 (SHELXL-2014/6).[2] The non-hydrogen atoms were refined with anisotropic displacement parameters without any constraints. The H atoms of the methyl groups were refined with common isotropic displacement parameters for the H atoms of the same group and idealized geometries with tetrahedral angles, enabling rotations around the C-S bonds and C-C bonds, and C-H distances of 0.98 Å. The H atoms of the CH_2 group were refined with common isotropic displacement parameters and idealized geometry with approximately tetrahedral angles and C-H distances of 0.99 Å. The H atoms of the phenyl ring were put at the external bisectors of the C-C-C angles at C-H distances of 0.95 Å and a common isotropic displacement parameter was refined for these H atoms. For complex **3**, since twinning was detected, an appropriate twin matrix (-1 0 0 / 0 -1 0 / 0 0 1) was applied with a BASF factor of 0.0269(4) between the two unequal twin components.

The X-ray data collections for complexes **1**, **2,** and **4** were performed with a XtaLAB Synergy, Dualflex, HyPix-Arc 100 diffractometer at 100 K with Cu Kα radiation (λ= 1.54184 Å) for **1** and **2** and with Mo Kα radiation (λ= 0.71073 Å) for **4**. Data reduction, scaling and

absorption corrections were performed using the *CrysAlisPro* software.[3] A numerical absorption correction based on gaussian integration over a multifaceted crystal model and an empirical absorption correction using spherical harmonics, implemented in SCALE3 ABSPACK scaling algorithm, were performed. The structures were solved with the ShelXT 2018/2[4] solution program using the intrinsic phasing solution method and by using the Olex2 software[5] as the graphical interface. The models were refined with ShelXL 2019/3[2] using full matrix least squares minimization on F^2. All non-hydrogen atoms were refined anisotropically. Hydrogen atom positions were calculated geometrically and refined using a riding model. For complex **1**, DFIX, DANG, SADI, RIGU and SIMU constraints and restraints were used to model the disorder at one of the *tert*-butyl group and model the cyclohexane solvent molecule. For complex **2**, since twinning was detected, an appropriate twin matrix (-1 0 0 / 0 -1 0 / 0 0 1) was applied with a BASF factor of 0.1062(19) between the two unequal twin components. SADI, RIGU, SIMU constraints were used to model the disorder of the *tert*-butyl ligands. DFIX, DANG, FLAT, SADI, RIGU and SIMU constraints and restraints were used to model one of the phenyl groups of the phosphine ligand. The absolute structure determination was obtained by anomalous dispersion. Since the hexane solvent molecule was highly disordered, the electron densities were removed from the electron density map by the SQUEEZE treatment using the OLEX2 solvent mask command. A solvent mask was calculated and 96 electrons were found in a volume of 555 $Å^3$ in 1 void per unit cell. This is consistent with the presence of 1 molecule hexane per unit cell (Z= 4), which account for 100 electrons.

CCDC 1913785 (complex **P1**), 1913784 (complex **P1′**), 2500256 (complex **1**), 2500257 (complex **2**), 1913788 (complex **3**) and 2500259 (complex **4**) contain the supplementary crystallographic data for this paper. This data can be obtained free of charge via http://www.ccdc.cam.ac.uk/ or from Cambridge Crystallographic Data Centre, 12 Union Road, Cambridge, CB2 1EZ, UK.

Table S1.Crystal data and structure refinement for **P1**, **P1′** and **1**.

Compound	**P1**	**P1′**	**1**
CCDC n°	1913785	1913784	2500256
Identification code	JS79E	JS79D	AD32
Empirical formula	$C_4H_{12}Cl_3OReS_2$	$C_8H_{24}Cl_4O_3Re_2S_4$	$C_{38}H_{56}Cl_4N_2O_7Re_2$ • C_6H_{12}
Formula weight	432.81	810.71	1251.20
Crystal system	monoclinic	monoclinic	monoclinic
Space group	$P2_1/c$	C2/c	$P2_1/c$
a /Å	7.8683(5)	17.0471(11)	15.0500(2)
b /Å	12.6536(8	8.4619(5)	19.9825(2)
c /Å	12.0886(7)	16.6401(9)	16.4972(2)
α /°	90	90	90
β /°	103.407(2)	116.328(5)	100.3220(10)
γ /°	90	90	90
Volume /Å^3	1170.77(13)	2151.4(2)	4881.02(10)
Z	4	4	4
ρ_{calc} g/cm^3	2.455	2.503	1.703
μ /mm^{-1}	11.372	12.131	11.951
F(000)	808	1512	2480.0
Crystal size /mm^3	0.26 × 0.16 × 0.13	0.24× 0.13 × 0.05	0.14 × 0.11 × 0.07
Radiation	Mo Kα (λ=0.71073 Å)	Mo Kα (λ=0.71073 Å)	Cu Kα (λ = 1.54184 Å)
2Θ range for data collection /°	4.52 to 80.00	5.34 to 70.00	5.97 to 154.758
Reflections collected	54046	24902	54132
Independent reflections	6499 [R_{int} = 0.1070, R_{sigma}= 0.0450]	3316 [R_{int} = 0.0666, R_{sigma}= 0.0606]	10338 [R_{int}=0.0517, R_{sigma}= 0.0359]
Data / restraints / parameters	7253 / 0 / 108	4744 / 0 / 104	10338 / 116 / 579
Goodness-of-fit on F^2	1.045	1.071	1.038
Final R indexes [I ≥ 2σ(I)]	R_1 = 0.0267, wR_2 = 0.0668	R_1 = 0.0310, wR_2 = 0.0802	R_1 = 0.0386, wR_2 = 0.1024
Final R indexes [all data]	R_1 = 0.0316, wR_2 = 0.0694	R_1 = 0.0515, wR_2 = 0.0883	R_1 = 0.0409, wR_2 = 0.1039
Largest diff. peak/hole / e.Å^{-3}	1.984 / -2.084	2.237 / -1.829	1.90 / -1.78

Table S2. Crystal data and structure refinement for **2**, **3** and **4**.

Compound	**2**	**3**	**4**
CCDC n°	2500257	1913788	2500259
Identification code	AD99-2	JSGO10B	AD124
Empirical formula	$C_{40}H_{50}Cl_2NO_3PRe$	$C_{19}H_{28}Cl_2NO_4Re$	$C_{21}H_{34}NO_3SCl_2Re$
Formula weight	880.88	591.52	637.65
Crystal system	monoclinic	monoclinic	triclinic
Space group	$P2_1$	$P2_1/n$	P-1
a /Å	16.28730(10)	12.1609(14)	10.0035(2)
b /Å	10.78790(10)	9.9571(13)	11.1092(2)
c /Å	22.2775(2)	18.691(2)	12.0885(3)
α /°	90	90	67.984(2)
β /°	91.2780(10)	103.208(7)	83.086(2)
γ /°	90	90	89.813(2)
Volume /$Å^3$	3913.31(6)	2203.4(5)	1235.11(5)
Z	4	4	2
ρ_{calc} g/cm^3	1.495	1.783	1.715
μ /mm^{-1}	8.001	5.780	5.241
F(000)	1780.0	1160	632.0
Crystal size /mm^3	0.12 × 0.08 × 0.02	0.29 × 0.06 × 0.02	0.20 × 0.09 × 0.08
Radiation	Cu Kα (λ = 1.54184 Å)	Mo Kα (λ = 0.71073 Å)	Mo Kα (λ = 0.71073 Å)
2Θ range for data collection /°	5.428 to 148.962	3.44 to 60.00	5.574 to 61.016
Reflections collected	15976	23806	36927
Independent reflections	15976 [R_{int} = -, R_{sigma} = 0.0381]	5402 [R_{int} = 0.0587, R_{sigma} = 0.0608]	7537 [R_{int} = 0.0326, R_{sigma} = 0.0283]
Data / restraints / parameters	15976 / 255 /890	6410 / 0 / 263	7537 / 0 / 272
Goodness-of-fit on F^2	1.051	1.011	1.206
Final R indexes [I ≥ 2σ(I)]	R_1 = 0.0474, wR_2 = 0.1232	R_1 = 0.0446, wR_2 = 0.1151	R_1 = 0.0257,wR_2 = 0.0523
Final R indexes [all data]	R_1 = 0.0506, wR_2 = 0.1255	R_1 = 0.0534, wR_2 = 0.1187	R_1 = 0.0301, wR_2 = 0.0531
Largest diff. peak/hole / e.$Å^{-3}$	2.78 / -1.59	1.581 / -1.517	1.62 / -1.94

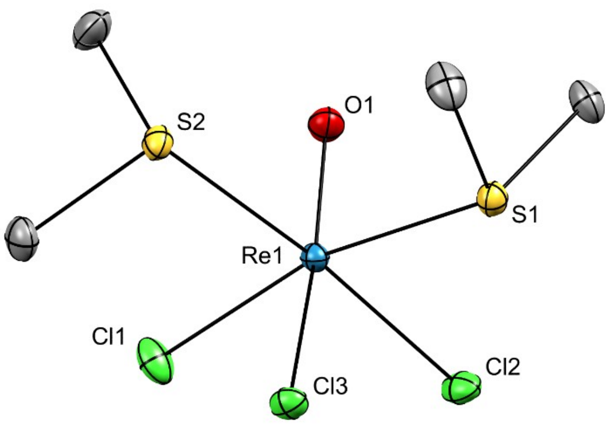

Fig. S10. Molecular structure of **P1**. The probability ellipsoids are drawn at 50%. The H atoms were omitted for clarity.

Table S3. Selected bond lengths in Å for **P1**.

Atom	Atom	Length/Å
Re1	O1	1.6737(18)
Re1	Cl1	2.3682(6)
Re1	Cl2	2.3619(6)
Re1	Cl3	2.4048(5)
Re1	S1	2.4410(6)
Re1	S2	2.5014(6)

Table S4. Selected bond angles in ° for **P1**.

Atom	Atom	Atom	Angle/°
O1	Re1	Cl1	100.58(7)
O1	Re1	Cl2	102.40(7)
O1	Re1	Cl3	162.38(7)
Cl1	Re1	Cl2	88.41(2)
Cl2	Re1	Cl3	92.05(2)
Cl1	Re1	Cl3	89.71(2)
O1	Re1	S1	91.37(7)
O1	Re1	S2	85.44(7)

Atom	Atom	Atom	Angle/°
Cl1	Re1	S1	166.99(2)
Cl2	Re1	S1	83.98(2)
Cl3	Re1	S1	80.06(2)
Cl1	Re1	S2	91.33(2)
Cl2	Re1	S2	172.07(2)
Cl3	Re1	S2	80.02(2)
S1	Re1	S2	94.76(2)

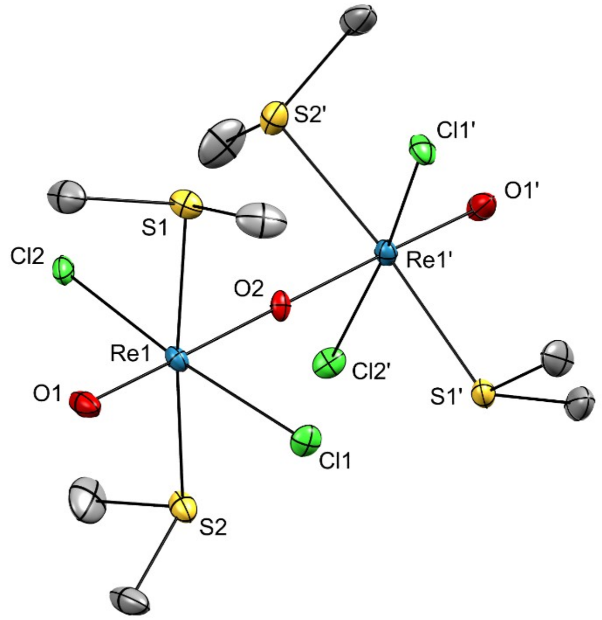

Fig. S11. Molecular structure of **P1′**. The probability ellipsoids are drawn at 50%. The H atoms were omitted for clarity. Symmetry transformation used to generate the equivalent atom: 1-x, y, 3/2-z.

Table S5. Selected bond lengths in Å for **P1′**.

**Atom**	**Atom**	**Length/Å**
Re1	O1	1.699(3)
Re1	O2	1.90911(17)
Re1	Cl1	2.4283(9)
Re1	Cl2	2.4603(10)
Re1	S1	2.4533(10)
Re1	S2	2.4535(11)

Table S6. Selected bond angles in ° for **P1′**.

**Atom**	**Atom**	**Atom**	**Angle/°**	**Atom**	**Atom**	**Atom**	**Angle/°**
Re1	O2	Re1′	100.58(7)	C12	S1	Re1	91.37(7)
O1	Re1	O2	102.40(7)	C11	S1	C12	85.44(7)
S1	Re1	S2	162.38(7)	C21	S2	Re1	166.99(2)
Cl1	Re1	Cl2	88.41(2)	C22	S2	Re1	83.98(2)
C11	S1	Re1	89.71(2)	C21	S2	C22	80.06(2)

Symmetry transformation used to generate the equivalent atom: 1-x, y, 3/2-z.

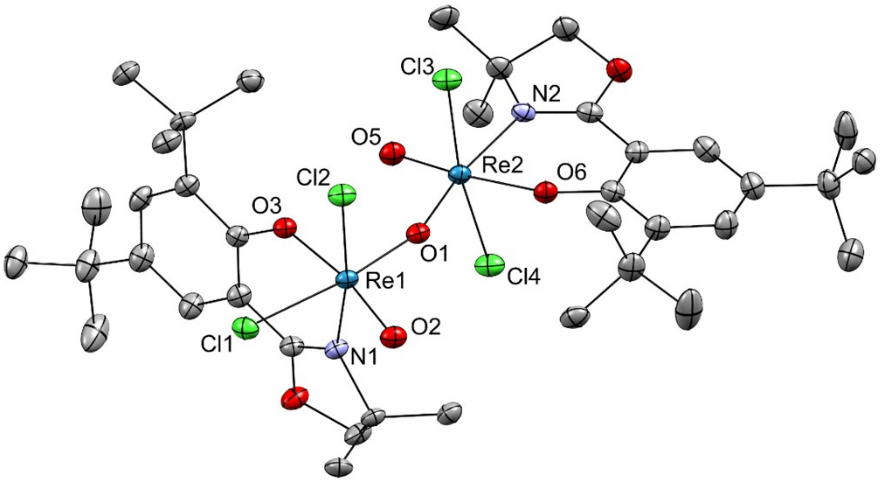

Fig. S12. Molecular structure of the complex **1**. The probability ellipsoids are drawn at 50%. the H atoms and the cyclohexane solvent molecule were omitted for clarity.

Table S7. Selected bond lengths in Å for **1**.

Atom	Atom	Length/Å
Re1	Cl1	2.3828(11)
Re1	Cl2	2.3192(10)
Re1	O1	1.869(3)
Re1	O2	1.684(3)
Re1	O3	1.933(3)
Re1	N1	2.113(4)
Re2	Cl3	2.3445(12)
Re2	Cl4	2.3793(11)
Re2	O1	1.845(3)
Re2	O5	1.688(3)
Re2	O6	1.922(3)

Atom	Atom	Length/Å
Re2	N2	2.143(4)
O3	C1	1.329(5)
O4	C7	1.348(5)
O4	C8	1.449(6)
O6	C21	1.334(6)
O7	C27	1.334(6)
O7	C28	1.438(6)
N1	C7	1.307(6)
N1	C9	1.525(6)
N2	C27	1.298(6)
N2	C29	1.507(6)

Table S8. Selected bond angles in ° for **1.**

Atom	Atom	Atom	Angle/°
Re1	O1	Re2	157.91(19)
Cl1	Re1	Cl2	89.13(4)
O1	Re1	Cl1	171.73(10)
O1	Re1	Cl2	90.67(10)
O1	Re1	O3	87.34(14)
O1	Re1	N1	92.10(14)
O2	Re1	Cl1	89.52(12)
O2	Re1	Cl2	98.42(11)
O2	Re1	O1	98.68(15)
O2	Re1	O3	169.13(14)
O2	Re1	N1	89.90(15)
O3	Re1	Cl1	84.40(10)
O3	Re1	Cl2	90.50(9)
O3	Re1	N1	80.80(14)
N1	Re1	Cl1	86.86(11)
N1	Re1	Cl2	170.73(11)

Atom	Atom	Atom	Angle/°
Cl3	Re2	Cl4	171.38(5)
O1	Re2	Cl3	93.45(10)
O1	Re2	Cl4	92.29(10)
O1	Re2	O6	89.26(14)
O1	Re2	N2	170.49(14)
O5	Re2	Cl3	94.14(12)
O5	Re2	Cl4	91.18(12)
O5	Re2	O1	100.07(16)
O5	Re2	O6	170.21(16)
O5	Re2	N2	89.22(16)
O6	Re2	Cl3	88.17(10)
O6	Re2	Cl4	85.47(10)
O6	Re2	N2	81.36(14)
N2	Re2	Cl3	87.82(11)
N2	Re2	Cl4	85.48(11)

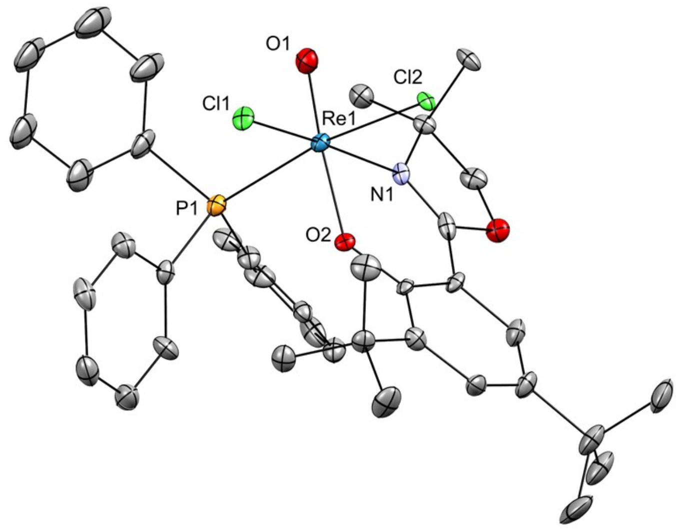

Fig. S13. Molecular structure of one complex of **2**. The probability ellipsoids are drawn at 30%. The H atoms and solvent molecule were omitted for clarity. For the disordered tert-butyl group, only the positions with the highest occupancies are depicted.

Table S9. Selected bond lengths in Å for **2**.

Atom	Atom	Length/Å	Atom	Atom	Length/Å
Re1	Cl1	2.366(3)	Re2	Cl11	2.368(3)
Re1	Cl2	2.438(2)	Re2	Cl12	2.446(2)
Re1	P1	2.490(3)	Re2	P11	2.504(3)
Re1	O1	1.681(7)	Re2	O11	1.691(7)
Re1	O2	1.963(6)	Re2	O12	1.947(6)
Re1	N1	2.160(9)	Re2	N11	2.147(9)
O2	C1	1.353(11)	O12	C41	1.340(12)

Table S10. Selected bond angles in ° for **2**.

Atom	Atom	Atom	Angle/°	Atom	Atom	Atom	Angle/°
Cl1	Re1	Cl2	88.56(9)	O11	Re2	Cl11	96.3(3)
Cl1	Re1	P1	90.95(9)	O11	Re2	Cl12	94.9(3)
Cl2	Re1	P1	170.45(10)	O11	Re2	P11	95.2(3)
O1	Re1	Cl1	96.7(3)	O11	Re2	O12	171.7(4)
O1	Re1	Cl2	95.7(3)	O11	Re2	N11	90.7(4)
O1	Re1	P1	93.8(3)	O12	Re2	Cl11	91.9(2)
O1	Re1	O2	171.4(4)	O12	Re2	Cl12	84.7(2)
O1	Re1	N1	90.3(4)	O12	Re2	P11	85.2(2)
O2	Re1	Cl1	91.8(2)	O12	Re2	N11	81.1(3)
O2	Re1	Cl2	85.2(2)	N11	Re2	Cl11	171.4(3)
O2	Re1	P1	85.2(2)	N11	Re2	Cl12	85.9(2)
O2	Re1	N1	81.3(3)	N11	Re2	P11	92.5(3)
N1	Re1	Cl1	171.5(3)	C41	O12	Re2	138.5(6)
N1	Re1	Cl2	86.0(2)	C47	N11	Re2	126.0(7)
N1	Re1	P1	93.3(3)				
C1	O2	Re1	137.0(6)				
C7	N1	Re1	125.9(8)				
Cl11	Re2	Cl12	88.54(10)				
Cl11	Re2	P11	91.76(9)				
Cl12	Re2	P11	169.80(10)				

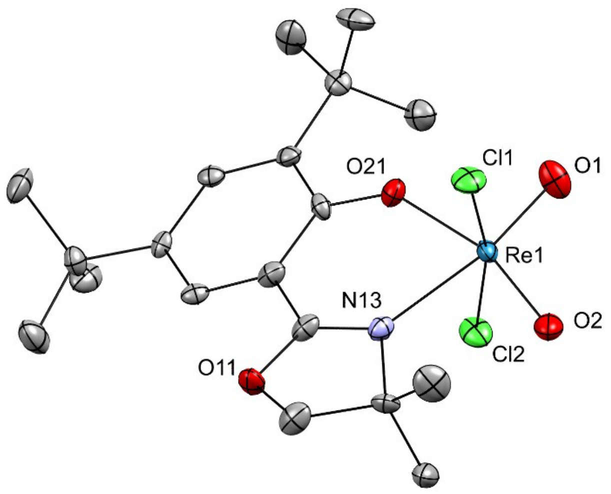

Fig. S14. Molecular structure of **3**. The probability ellipsoids are drawn at 50%. The H atoms were omitted for clarity.

Table S11. Selected bond lengths in Å for **3**.

Atom	Atom	Length/Å
Re1	O1	1.713(4)
Re1	O2	1.716(4)
Re1	O21	1.947(4)
Re1	N13	2.237(5)
Re1	Cl1	2.3280(13)
Re1	Cl2	2.3631(13)

Table S12. Selected bond angles in ° for **3**.

Atom	Atom	Atom	Angle/°
O1	Re1	O2	105.1(2)
O1	Re1	N13	169.48(19)
O2	Re1	O21	162.44(18)
Cl1	Re1	Cl2	167.46(5)
C12	N13	C14	105.5(5)
C12	N13	Re1	127.3(4)
C14	N13	Re1	126.5(3)
C21	O21	Re1	140.9(4)

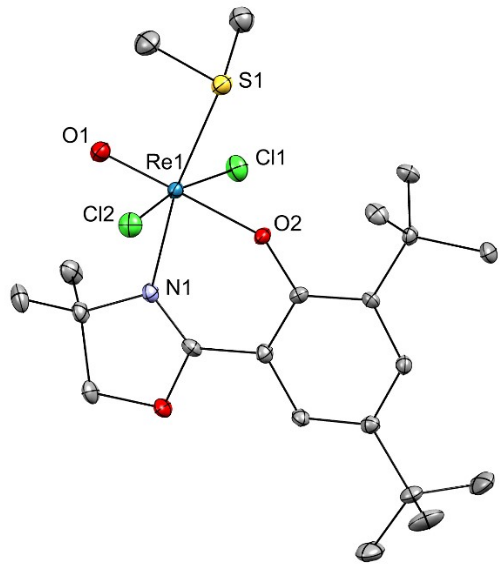

Fig. S15. Molecular structure of **4**. The probability ellipsoids are drawn at 50%. The H atoms were omitted for clarity.

Table S13. Selected bond lengths in Å for **4**.

Atom	Atom	Length/Å
Re1	Cl1	2.4149(8)
Re1	Cl2	2.3962(7)
Re1	S1	2.4128(8)
Re1	O1	1.691(2)
Re1	O2	1.929(2)
Re1	N1	2.128(2)

Table S14. Selected bond angles in ° for **4**.

Atom	Atom	Atom	Angle/°
Cl2	Re1	Cl1	170.48(3)
Cl2	Re1	S1	88.34(3)
S1	Re1	Cl1	93.23(3)
O1	Re1	Cl1	92.54(8)
O1	Re1	Cl2	96.67(8)
O1	Re1	S1	95.34(7)
O1	Re1	O2	175.86(10)
O1	Re1	N1	95.33(10)
O2	Re1	Cl1	83.96(7)
O2	Re1	Cl2	86.74(7)
O2	Re1	S1	87.07(6)
O2	Re1	N1	82.39(9)

Atom	Atom	Atom	Angle/°
N1	Re1	Cl1	88.24(7)
N1	Re1	Cl2	88.49(7)
N1	Re1	S1	169.15(7)
C20	S1	Re1	105.58(12)
C20	S1	C21	99.94(17)
C21	S1	Re1	109.35(11)
C1	O2	Re1	141.24(18)
C7	O3	C8	107.7(2)
C7	N1	Re1	126.58(19)
C7	N1	C9	109.5(2)
C9	N1	Re1	123.91(18)

Computational Details

All DFT calculations were performed with TURBOMOLE 7.8.[6–10] Geometries were optimized using both the PBE[11] and B3LYP[12–14] density functional approximations, together with the D3BJ dispersion correction[15,16] and the dhf-SVP basis set.[17] Convergence criteria for the geometries were set to 10^{-7} Hartree and 10^{-4} Hartree/bohr. The SCF was converged to 10^{-9} Hartree.

Solvent effects were included by employing the COSMO solvation model for cyclohexane ($\varepsilon_r = 2.023$).[18] To account for relativistic effects at the rhenium center, the corresponding dhf-ECP (effective core potential) was used.[19] To accelerate the calculations, the resolution-of-identity approximation (RI-J for PBE; RI-JK for B3LYP) was applied.[20–24] Analytical normal modes were determined using TURBOMOLE's *aoforce* program to confirm the stationary points. The rigid-rotor–harmonic-oscillator (RRHO) approximation was used to calculate zero-point vibrational energies and thermal properties at room temperature (298.15 K).

For both employed functionals, dhf-TZVPP[17] single-point calculations were performed at the respective dhf-SVP geometries, again using the RI-J/RI-JK approximation and the COSMO solvation model. Zero-point energies and thermal corrections for the single-point calculations were taken from the dhf-SVP results. The data reported in the main text are the B3LYP-D3BJ/dhf-TZVPP+COSMO single-point results, unless otherwise noted.

Computational Data

A comparison of the calculated Gibbs free energies (ΔG) obtained from the different computational methods used for Fig. 7 shows that the variations between the methods are minimal. All approaches yield consistent relative energetics, and no method deviates in a way that would affect the qualitative interpretation of the results. Since the differences are negligible, the computationally most reliable and best-performing method was chosen for the data presented in the main text.

	dhf-SVP		***dhf-TZVPP***	
	**PBE-D3BJ**	**B3LYP-D3BJ**	**PBE-D3BJ**	**B3LYP-D3BJ**
***P1**$_{fac}$*	0.0	0.0	0.0	0.0
***P1**$_{mer\text{-}trans}$*	-1.29	-4.13	3.42	-3.04
***P1**$_{mer\text{-}cis}$*	7.78	4.07	6.51	4.13
P1**$_{fac}$ to **4	-77.40	-87.39	-72.84	-82.02
P1**$_{fac}$ to **5	-73.64	-83.44	-67.95	-78.39
***P1**$_{fac}$ to* all-*cis* complex **6**	-134.57	-109.34	-128.55	-105.31
***P1**$_{fac}$ to* all-trans complex *7*	-173.12	-151.92	-168.32	-148.49
P1**$_{fac}$ to Deep Purple **1	-188.94	-171.32	-179.63	-163.23

Table S 15: Comparison of computational methods in the gas phase (**P1**$_{fac}$ = 0).

Table S 16: Comparison of computational methods for cyclohexane using the COSMO model (cyclohexane)

	dhf-SVP		*dhf-TZVPP*	
	**PBE-D3BJ**	**B3LYP-D3BJ**	**PBE-D3BJ**	**B3LYP-D3BJ**
***P1**$_{fac}$*	0.0	0.0	0.0	0.0
***P1**$_{mer\text{-}trans}$*	-5.60	2.78	-5.43	3.24
***P1**$_{mer\text{-}cis}$*	9.15	12.93	7.76	12.40
P1**$_{fac}$ to **4	-70.35	-82.26	-68.33	-76.75
P1**$_{fac}$ to **5	-69.92	-75.59	-61.21	-70.34
***P1**$_{fac}$ to* all-cis complex **6**	-124.48	-96.78	-118.06	-92.38
***P1**$_{fac}$ to* all-trans complex *7*	-165.49	-141.34	-160.25	-137.42
P1**$_{fac}$ to Deep Purple **1	-178.76	-158.84	-169.12	-150.38

(**P1**$_{fac}$ = 0).

Table S 17: Raw energies calculated using PBE-D3BJ/dhf-SVP.

Species	*Chem. Pot. [kj/mol]*	*total energy [Eh]*
*Deep purple **1***	2030.45	-4111.10673021534
*Complex **6***	2034.77	-4111.06696351256
Complex 7	2025.14	-4111.09265618960
***P1**$_{fac}$*	289.59	-2488.66787267363
***P1**$_{mer\text{-}trans}$*	285.99	-2488.66699128088
***P1**$_{mer\text{-}cis}$*	286.36	-2488.66367916551
DMS	121.09	-477.53883274963
O_2	-41.90	-150.06614917101
HCl	-29.73	-460.46167677702
*Complex **5***	1158.94	-2495.53637393700
*Complex **4***	1155.19	-2495.53637783226
Ligand	998.81	-944.85547391792

Table S 18: Raw energies calculated using PBE-D3BJ/dhf-TZVPP.

Species	*Chem. Pot. [kj/mol]*	*total energy [Eh]*
*Deep purple **1***	2030.45	-4114.08207605257
*Complex **6***	2034.77	-4114.04481095155
Complex 7	2025.14	-4114.07144041928
$\mathbf{P1}_{fac}$	289.59	-2489.71858208313
$\mathbf{P1}_{mer\text{-}trans}$	285.99	-2489.71590644835
$\mathbf{P1}_{mer\text{-}cis}$	286.36	-2489.71487268819
DMS	121.09	-477.77234153750
O_2	-41.90	-150.24918232542
HCl	-29.73	-460.62780979714
*Complex **5***	1158.94	-2497.21317633768
*Complex **4***	1155.19	-2497.21360999328
Ligand	998.81	-945.88337677877

Table S 19: Raw energies calculated at the PBE-D3BJ/dhf-SVP level with COSMO (cyclohexane).

Species	*Chem. Pot. [kj/mol]*	*total energy [Eh]*
*Deep purple **1***	2027.24	-4111.11487163125
*Complex **6***	2032.51	-4111.07553455797
Complex 7	2021.03	-4111.10239603810
$\mathbf{P1}_{fac}$	287.11	-2488.67468148711
$\mathbf{P1}_{mer\text{-}trans}$	281.34	-2488.67461584282
$\mathbf{P1}_{mer\text{-}cis}$	282.81	-2488.66956017676
DMS	120.65	-477.54051193264
O_2	-41.89	-150.06631064535
HCl	-29.81	-460.46357899885
*Complex **5***	1156.20	-2495.54427806121
*Complex **4***	1152.89	-2495.54318307433
Ligand	996.01	-944.86079983172

Table S 20: Raw energies calculated using PBE-D3BJ/dhf-TZVPP with COSMO (cyclohexane).

Spezies	*Chem. Pot. [kj/mol]*	*total energy [Eh]*
*Deep purple **1***	2027.24	-4114.09070967171
*Complex **6***	2032.51	-4114.05382086932
Complex 7	2021.03	-4114.08159002312
***P1**$_{fac}$*	287.11	-2489.72561538041
***P1**$_{mer-trans}$*	281.34	-2489.72548671049
***P1**$_{mer-cis}$*	282.81	-2489.72102378834
DMS	120.65	-477.77419364649
O_2	-41.89	-150.24930372111
HCl	-29.81	-460.62963701844
*Complex **5***	1156.20	-2497.22048539737
*Complex **4***	1152.89	-2497.22193668285
Ligand	996.01	-945.50022303521

Table S 21: Raw energies obtained with the B3LYP-D3BJ/dhf-SVP method.

Species	*Chem. Pot. [kj/mol]*	*total energy [Eh]*
*Deep purple **1***	2098.89	-4112.87074503373
*Complex **6***	2103.47	-4112.82527441349
Complex 7	2094.04	-4112.85411614015
***P1**$_{fac}$*	302.47	-2489.41042920197
***P1**$_{mer-trans}$*	298.32	-2489.41042255581
***P1**$_{mer-cis}$*	298.63	-2489.40741702212
DMS	125.99	-477.72780469435
O_2	-41.33	-150.14528506114
HCl	-29.45	-460.60165482920
*Complex **5***	1197.92	-2496.59800604712
*Complex **4***	1194.69	-2496.59827934269
Ligand	1031.27	-944.85547391792

Table S 22: Raw energies obtained with the B3LYP-D3BJ/dhf-TZVPP method.

Species	***Chem. Pot. [kj/mol]***	***total energy [Eh]***
*Deep purple **1***	2098.89	-4115.90054079690
*Complex **6***	2103.47	-4115.85816826261
Complex 7	2094.04	-4115.88746347412
$\mathbf{P1}_{fac}$	302.47	-2490.46912186700
$\mathbf{P1}_{mer\text{-}trans}$	298.32	-2490.46890804103
$\mathbf{P1}_{mer\text{-}cis}$	298.63	-2490.46632004093
DMS	125.99	-477.96399496868
O_2	-41.33	-150.33029543215
HCl	-29.45	-460.76785838087
*Complex **5***	1197.92	-2498.30400752021
*Complex **4***	1194.69	-2498.30416066927
Ligand	1031.27	-946.55184196485

Table S 23: Raw energies obtained with B3LYP-D3BJ/dhf-SVP using COSMO solvation (cyclohexane).

Species	***Chem. Pot. [kj/mol]***	***total energy [Eh]***
*Deep purple **1***	2095.29	-4112.87903059459
*Complex **6***	2101.03	-4112.83394465676
Complex 7	2091.18	-4112.86413976510
$\mathbf{P1}_{fac}$	300.10	-2489.41772356876
$\mathbf{P1}_{mer\text{-}trans}$	302.74	-2489.41766945595
$\mathbf{P1}_{mer\text{-}cis}$	302.49	-2489.41370966002
DMS	125.60	-477.72944704326
O_2	-41.32	-150.14546233044
HCl	-29.45	-460.60352785673
*Complex **5***	1194.89	-2496.60520061371
*Complex **4***	1192.07	-2496.60666761894
Ligand	1027.67	-945.50564969047

Table S 24: Raw energies obtained with B3LYP-D3BJ/dhf-TZVPP using COSMO solvation (cyclohexane).

Species	***Chem. Pot. [kj/mol]***	***total energy [Eh]***
*Deep purple **1***	2095.29	-4115.90941971143
*Complex **6***	2101.03	-4115.86741831535
Complex 7	2091.18	-4115.89798114446
$\mathbf{P1}_{fac}$	300.10	-2490.47679179108
$\mathbf{P1}_{mer\text{-}trans}$	302.74	-2490.47656220030
$\mathbf{P1}_{mer\text{-}cis}$	302.49	-2490.47297890697
DMS	125.60	-477.96584294206
O_2	-41.32	-150.33042746334
HCl	-29.45	-460.76969118703
*Complex **5***	1194.89	-2498.31177055482
*Complex **4***	1192.07	-2498.31313700949
Ligand	1027.67	-946.55771019386

IR-Measurement

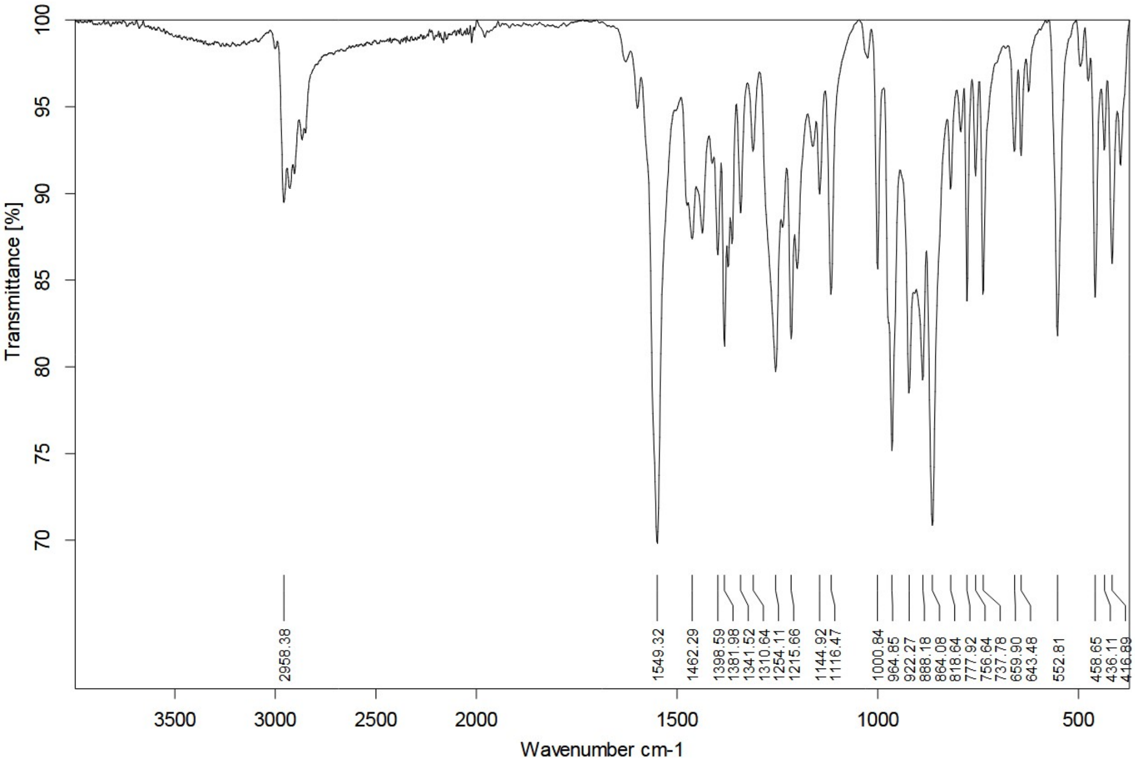

Fig. S16. IR spectrum of complex **1**

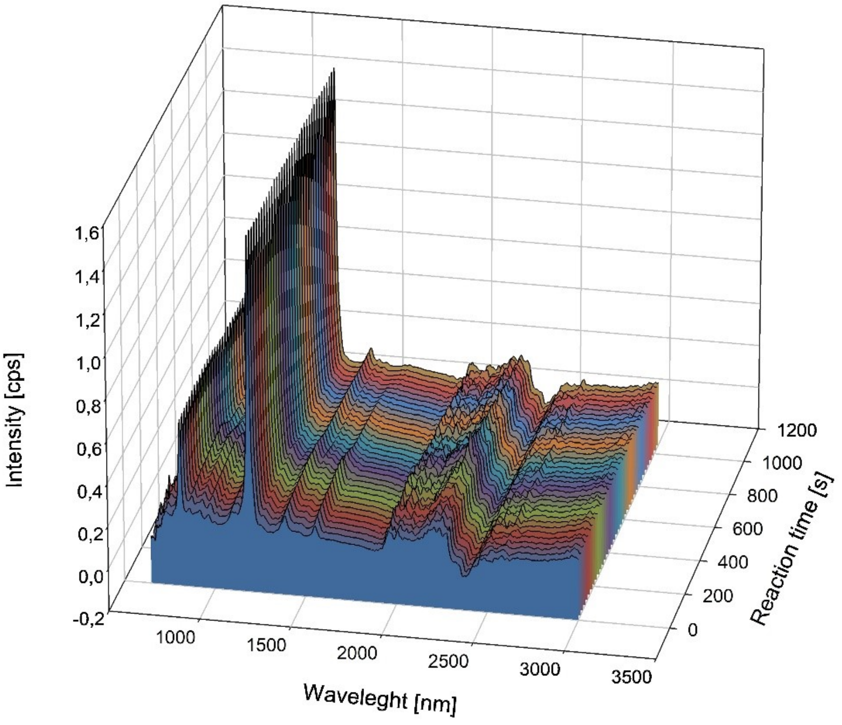

Fig. S17. *In-situ* IR measurement of the reaction of complex **1** with PPh_3

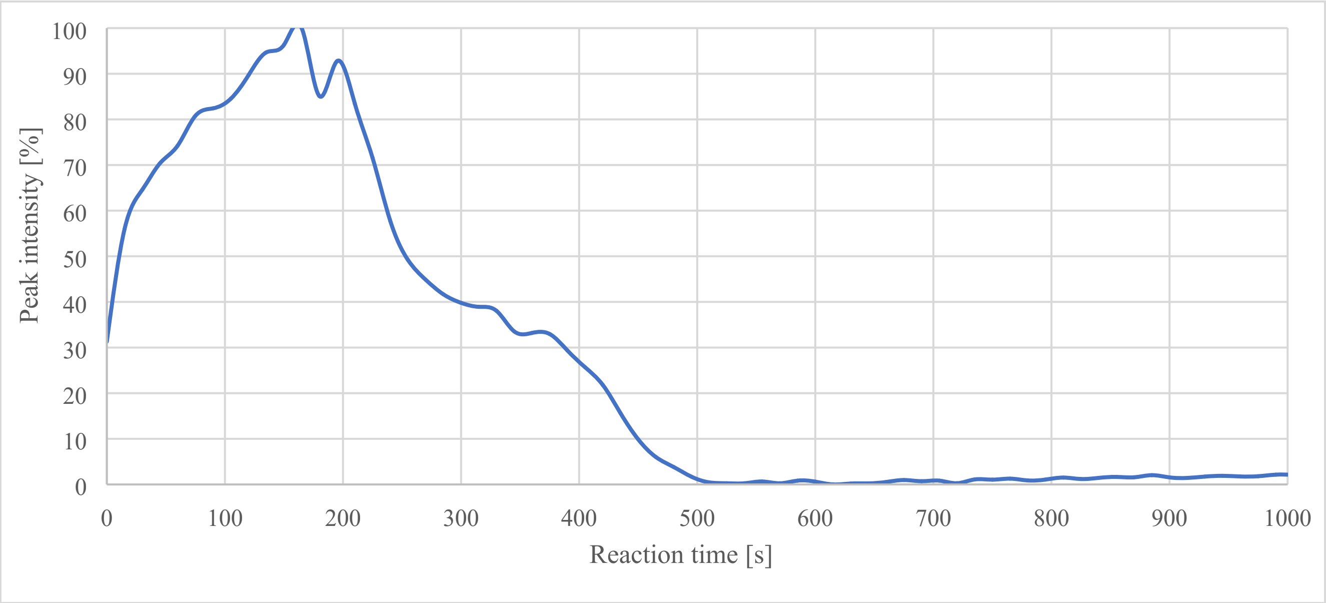

Fig. S18. IR peak intensity of PPh_3 at 1408 nm followed over time for the reaction of complex **1** with PPh_3

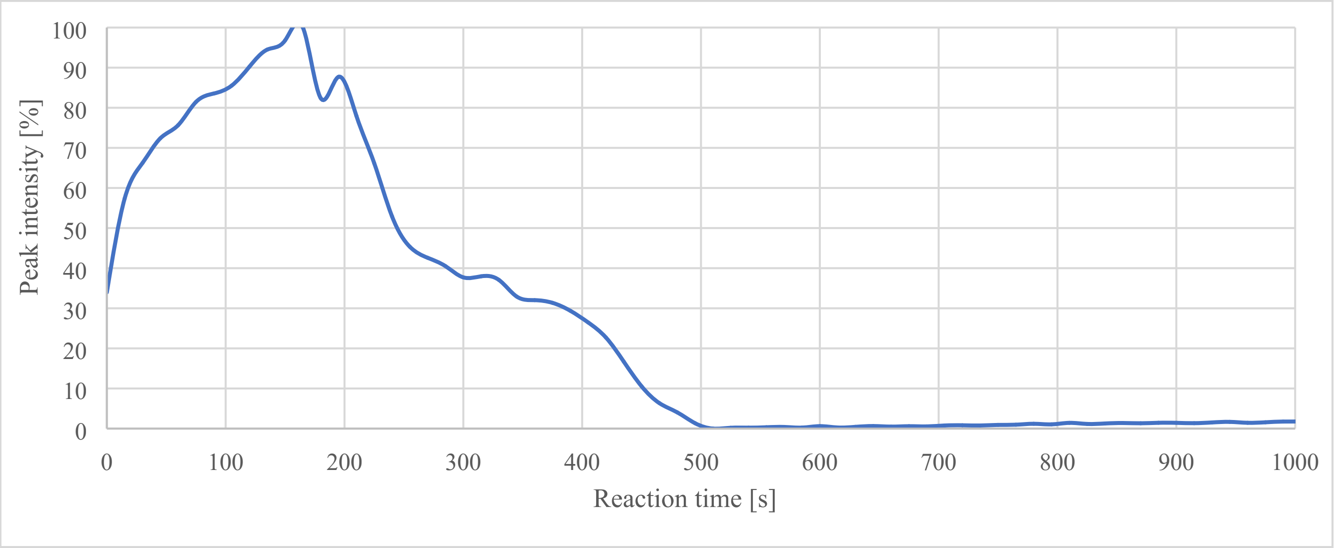

Fig. S19. IR peak intensity of PPh_3 at 960 nm followed over time for the reaction of complex **1** with PPh_3

UV-Vis Measurement

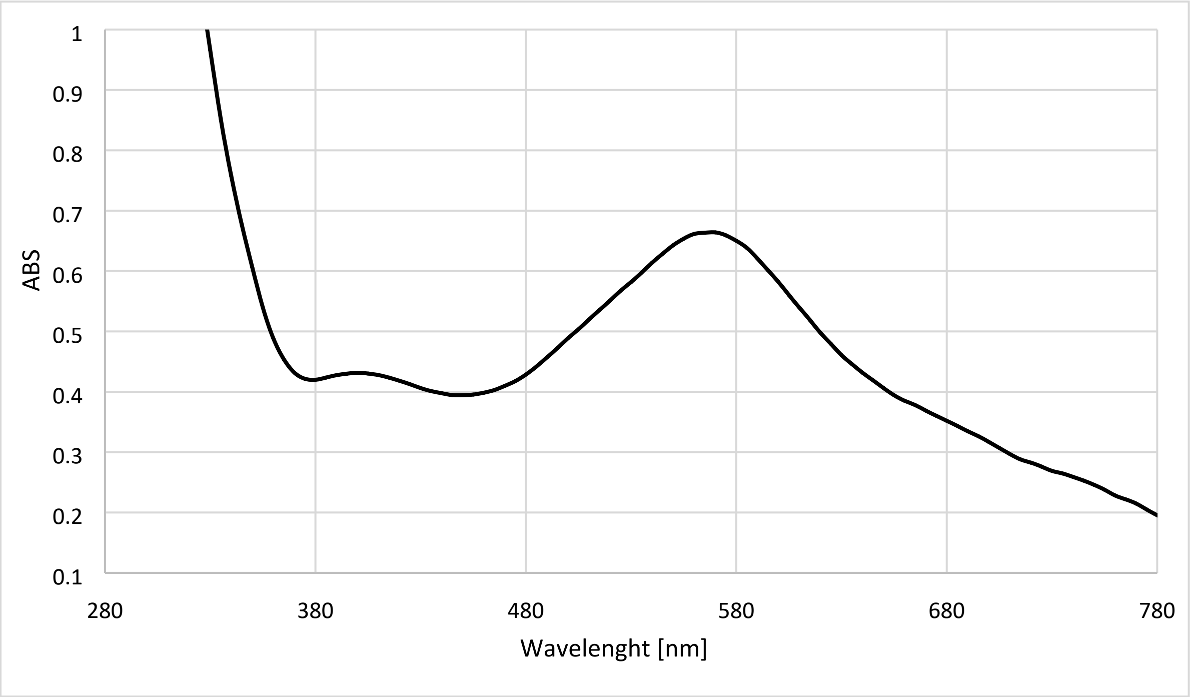

Fig. S20. UV-Vis measurement of a $CHCl_3$ solution (c = 0.0003 M) of complex **1**

References

1 G. M. Sheldrick, A short history of SHELX, *Acta Crystallogr. Sect. Found.*, 2008, 112–122.
2 G. M. Sheldrick, Crystal structure refinement with SHELXL, *Acta Crystallogr. Sect. C Struct. Chem.*, 2015, 3–8.
3 Rigaku Oxford Diffraction, CrysAlisPro, 2024.
4 G. M. Sheldrick, SHELXT – integrated space-group and crystal-structure determination, *Acta Crystallogr. Sect. Found.*, 2015, **71**, 3–8.
5 O. V. Dolomanov, L. J. Bourhis, R. J. Gildea, J. A. K. Howard and H. Puschmann, OLEX2: a complete structure solution, refinement and analysis program, *J. Appl. Crystallogr.*, 2009, **42**, 339–341.
6 *TURBOMOLE V7.8*, 2023.
7 O. Treutler and R. Ahlrichs, Efficient Molecular Numerical Integration Schemes, *J. Chem. Phys.*, 1995, **102**, 346–354.
8 O. Treutler and R. Ahlrichs, Efficient Molecular Numerical Integration Schemes, *J. Chem. Phys.*, 1995, **102**, 346.
9 M. von Arnim and R. Ahlrichs, Performance of Parallel TURBOMOLE for Density Functional Calculations, *J. Comput. Chem.*, 1998, **19**, 1746–1757.
10 R. Ahlrichs, M. Bär, M. Häser, H. Horn and C. Kölmel, Electronic structure calculations on workstation computers: The program system turbomole, *Chem. Phys. Lett.*, 1989, **162**, 165–169.
11 J. P. Perdew, K. Burke and M. Ernzerhof, Generalized Gradient Approximation Made Simple, *Phys. Rev. Lett.*, 1996, **77**, 3865–3868.
12 A. D. Becke, Density-Functional Exchange-Energy Approximation with Correct Asymptotic Behavior, *Phys. Rev. A*, 1988, **38**, 3098–3100.
13 A. D. Becke, Density-Functional Thermochemistry. III. The Role of Exact Exchange, *J. Chem. Phys.*, 1993, **98**, 5648–5652.
14 C. Lee, W. Yang and R. G. Parr, Development of the Colle–Salvetti Correlation-Energy Formula into a Functional of the Electron Density, *Phys. Rev. B*, 1988, **37**, 785–789.
15 S. Grimme, J. Antony, S. Ehrlich and H. Krieg, A Consistent and Accurate \emphab initio Parametrization of Density Functional Dispersion Correction (DFT-D) for the 94 Elements H–Pu, *J. Chem. Phys.*, 2010, **132**, 154104.
16 S. Grimme, S. Ehrlich and L. Goerigk, Effect of the Damping Function in Dispersion Corrected Density Functional Theory, *J. Comput. Chem.*, 2011, **32**, 1456–1465.
17 F. Weigend and A. Baldes, Segmented Contracted Basis Sets for One- and Two-Component Dirac–Fock Effective Core Potentials, *J. Chem. Phys.*, 2010, **133**, 174102.
18 A. Klamt and G. Schüürmann, COSMO: A New Approach to Dielectric Screening in Solvents with Explicit Expressions for the Screening Energy and Its Gradient, *J. Chem. Soc. Perkin Trans. 2*, 1993, 799–805.
19 D. Figgen, K. A. Peterson, M. Dolg and H. Stoll, Energy-Consistent Pseudopotentials and Correlation Consistent Basis Sets for the 5d Elements Hf–Pt, *J. Chem. Phys.*, 2009, **130**, 164108.
20 K. Eichkorn, O. Treutler, H. Öhm, M. Häser and R. Ahlrichs, Auxiliary Basis Sets to Approximate Coulomb Potentials, *Chem. Phys. Lett.*, 1995, **240**, 283–290.
21 K. Eichkorn, O. Treutler, H. Öhm, M. Häser and R. Ahlrichs, Auxiliary Basis Sets To Approximate Coulomb Potentials — ERRATUM, *Chem. Phys. Lett.*, 1995, **242**, 652–660.
22 K. Eichkorn, F. Weigend, O. Treutler and R. Ahlrichs, Auxiliary Basis Sets for Main Row Atoms and Transition Metals and Their Use To Approximate Coulomb Potentials, *Theor. Chem. Acc.*, 1997, **97**, 119–124.
23 F. Weigend, Accurate Coulomb-Fitting Basis Sets for H to Rn, *Phys. Chem. Chem. Phys.*, 2006, **8**, 1057–1065.

24 F. Weigend, A Fully Direct RI-HF Algorithm: Implementation, Optimised Auxiliary Basis Sets, Demonstration of Accuracy and Efficiency, *Phys. Chem. Chem. Phys.*, 2002, **4**, 4285–4291.
